# Reconstructing geographical parthenogenesis: effects of niche differentiation and reproductive mode on Holocene range expansion of an alpine plant

**DOI:** 10.1111/ele.12908

**Published:** 2018-01-19

**Authors:** Bernhard Kirchheimer, Johannes Wessely, Andreas Gattringer, Karl Hülber, Dietmar Moser, Christoph C. F. Schinkel, Marc Appelhans, Simone Klatt, Marco Caccianiga, Agnes Dellinger, Antoine Guisan, Michael Kuttner, Jonathan Lenoir, Luigi Maiorano, Diego Nieto‐Lugilde, Christoph Plutzar, Jens‐Christian Svenning, Wolfgang Willner, Elvira Hörandl, Stefan Dullinger

**Affiliations:** ^1^ Department of Botany and Biodiversity Research University of Vienna Rennweg 14 1030 Vienna Austria; ^2^ Department of Systematics, Biodiversity and Evolution of Plants (with herbarium) University of Goettingen Untere Karspüle 2 37073 Göttingen; ^3^ Department of Biosciences University of Milan Via Giovanni Celoria 26 20133 Milan Italy; ^4^ Department of Ecology & Evolution Biophore University of Lausanne 1015 Lausanne Switzerland; ^5^ Institute of Earth Surface Dynamics Geopolis University of Lausanne 1015 Lausanne Switzerland; ^6^ UR «Ecologie et Dynamique des Systèmes Anthropisés» (EDYSAN, FRE 3498 CNRS) Jules Verne University of Picardie 1 Rue des Louvels F‐80037 Amiens Cedex 1 France; ^7^ Department of Biology and Biotechnologies Sapienza University of Rome Viale dell'Università 32 Rome Italy; ^8^ Departamento de Botánica Ecología y Fisiología Vegetal Universidad de Córdoba 14071 Córdoba Spain; ^9^ Section for Ecoinformatics & Biodiversity Department of Bioscience Aarhus University Ny Munkegade 114‐116 8000 Aarhus C Denmark; ^10^ Vienna Institute for Nature Conservation and Analyses Gießergasse 6/7 1090 Vienna Austria

**Keywords:** Apomictic plants, European Alps, geographical range, minority cytotype disadvantage, niche shift, polyploidization, *Ranunculus kuepferi*

## Abstract

Asexual taxa often have larger ranges than their sexual progenitors, particularly in areas affected by Pleistocene glaciations. The reasons given for this ‘geographical parthenogenesis’ are contentious, with expansion of the ecological niche or colonisation advantages of uniparental reproduction assumed most important in case of plants. Here, we parameterized a spread model for the alpine buttercup *Ranunculus kuepferi* and reconstructed the joint Holocene range expansion of its sexual and apomictic cytotype across the European Alps under different simulation settings. We found that, rather than niche broadening or a higher migration rate, a shift of the apomict's niche towards colder conditions *per se* was crucial as it facilitated overcoming of topographical barriers, a factor likely relevant for many alpine apomicts. More generally, our simulations suggest potentially strong interacting effects of niche differentiation and reproductive modes on range formation of related sexual and asexual taxa arising from their differential sensitivity to minority cytotype disadvantage.

## Introduction

Asexual species often occupy larger geographical ranges than their sexual relatives and extend farther towards cold environments (e.g. Bierzychudek 1985; van Dijk 2003; Randle *et al*. 2009). This long‐recognised phenomenon has been coined ‘geographical parthenogenesis’ (Vandel 1928) and is puzzling because it suggests an advantage of asexuality that is at odds with the prevalence of sexual reproduction in animals and plants (Maynard Smith 1978; Kearney 2003; Schaefer *et al*. 2006). The processes underlying this biogeographic peculiarity are still contentious (e.g. Kearney 2005; Hörandl 2006, 2011). In plants, where asexual reproduction via seeds is called apomixis, two processes have been emphasised as particularly crucial to explain the success of apomictic lineages (Hörandl 2006): (1) a broadening of the ecological niche, and (2) uniparental reproduction.

In flowering plants, a switch to apomixis is commonly associated with the formation of polyploid cytotypes (Comai 2005). As niche differentiation has long been considered prerequisite to the establishment of polyploids (Levin 1975, 2003), apomictic (polyploid) taxa are expected to have environmental tolerances that are distinct from those of their diploid and sexual progenitors. In particular, their niches may not only differ but also be wider than those of the progenitors because polyploidization is often associated with a gain of genotypic diversity and genome flexibility (Comai 2005; Kearney 2005; Lowry & Lester 2006), or because recurrent establishment of asexual cytotypes from the same parent species can generate a swarm of ecologically distinct genotypes protected against erosion by gene flow (Frozen Niche Variation Model, Vrijenhoek 1984, 1994; Peck *et al*. 1998; Vrijenhoek & Parker 2009). However, this view has been challenged by recent studies suggesting that neither shifts of niche optima nor a broadening of the niche necessarily accompany the establishment of polyploid cytotypes (Theodoridis *et al*. 2013; Glennon *et al*. 2014; Kirchheimer *et al*. 2016).

An alternative line of reasoning focuses on the colonising advantages of uniparental reproduction: asexuality involves independence of mating partners. As a consequence, a long‐distance dispersal event for a single propagule is theoretically sufficient for the foundation of a new population ahead of a migrating front, causing Allee effects to be less influential in slowing range expansion (Baker's law, Stebbins 1950; Baker 1967). Indeed, self‐fertility has recently been shown to increase range size across a number of plant families (Grossenbacher *et al*. 2015). Most apomictic plant species are self‐fertile, but usually evolve from self‐incompatible diploid outcrossers (Hörandl 2010). Thus, uniparentally reproducing apomicts commonly have biparentally reproducing sexual relatives making Baker's law a plausible mechanism for the emergence of geographical parthenogenesis.

The pros and cons of explaining geographical parthenogenesis by these two underlying ecological processes have been discussed in several reviews (Kearney 2005; Hörandl 2006; Hörandl *et al*. 2008; Morgan‐Richards *et al*. 2010). In particular, Hörandl (2006) concluded that causality is probably complex and that determinants may interact with each other and with the environment. However, these considerations remain largely speculative. What is lacking so far is a study that explicitly evaluates the relative contribution of the two processes and their possible interaction with the emergence of range size differences in a particular pair or group of plant species.

**Figure 1:**
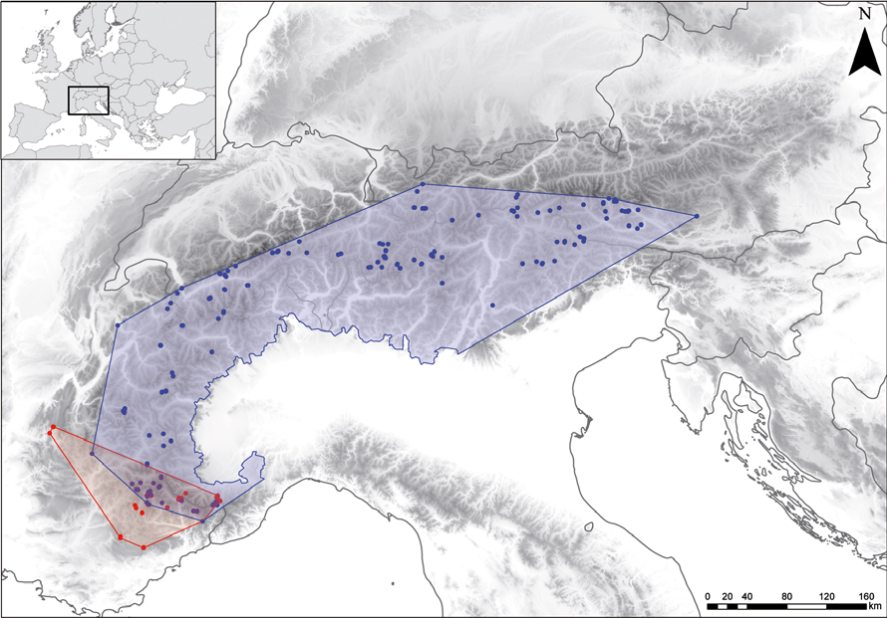
Current distribution of the two cytotypes of *Ranunculus kuepferi*. Red and blue dots represent sampled populations of diploids and tetraploids respectively. Surrounding convex hull areas are coloured correspondingly. Areas outside the Alpine chain were removed from the hulls.

Here, we undertake such an attempt based on simulations of the Holocene range dynamics of a diploid sexual plant and its tetraploid and apomictic relative. We focus on *Ranunculus kuepferi* Greuter & Burdet, an alpine buttercup with pronounced geographical parthenogenesis in the European Alps (Cosendai *et al*. 2013): diploid populations are restricted to the south‐western fringes of the Alps, while tetraploid populations extend over nearly the entire Alpine chain (see Fig. 1). In a previous study, we found evidence that tetraploids have niche optima that shifted towards colder conditions, whereas their niche breadth decreased rather than increased as compared to diploids (Kirchheimer *et al*. 2016). Here, we use a spatially and temporarily explicit plant spread model to simulate the Holocene range expansion of both cytotypes from their putative glacial refugia. For the simulations, we switch the cytotypes’ ecological niches and reproductive modes in a factorial design and account for possible minority cytotype disadvantages (Levin 1975) where populations occur in sympatry. This simulation experiment allows evaluating the relative importance of niche differentiation or uniparental reproduction in determining the pronounced range size difference between cytotypes and how these processes interacted with each other and with the changing environment.

## Material and Methods

### Species

*Ranunculus kuepferi* is a perennial herb growing in various types of grasslands at elevations between 1300 and 2800 m. The white flowers are insect‐pollinated and produce *c*. 5–30 achenes, which serve as diaspores (Müller‐Schneider 1986). For more details on species traits, see Table S1 in Supporting Information.

Diploid populations of *R. kuepferi* have survived the last glacial maximum (LGM) in spatially scattered sites at the south‐western fringes of the European Alps (Burnier *et al*. 2009). Molecular data suggested that the tetraploid cytotype has most likely emerged from repeated autopolyploidization (Cosendai *et al*. 2011, 2013). Here, we conducted molecular dating showing that tetraploids originated during the Late Pleistocene or the early Holocene, while diploids are much older (see Appendix S1 and Fig. S1).

While diploids are predominantly sexual outcrossers, tetraploids mainly reproduce by pseudogamous apomixis. Rare cases of apomictic diploids have been documented and tetraploids are, in principle, capable of sexual seed production. Nevertheless, diploid asexual and tetraploid sexual populations have never been observed in the field (Schinkel *et al*. 2016, 2017). Some populations in the restricted geographical contact zone are mixed, and triploid as well as pentaploid individuals occur (Cosendai & Hörandl 2010; Schinkel *et al*. 2016).

### Study area

We simulated the post‐glacial recolonisation of the European Alps by both sexual and asexual lineages of *R. kuepferi*. The study area was represented by a two‐dimensional raster with a spatial resolution of 100 × 100 m^2^ (individual cells of the raster are called sites henceforth).

Maps of the area's climate history (mean, minimum and maximum monthly temperatures and monthly precipitation sums) back to 10 kyr BP were taken from Maiorano *et al*. (2013), who had downscaled time series of global climate simulations by the HadCM3 atmosphere‐ocean general circulation model (Singarayer & Valdes 2010) to a spatial resolution of 100 × 100 m^2^ (see Espindola *et al*. 2012; Maiorano *et al*. 2013 for downscaling methods). We temporally downscaled the original 1‐kyr temporal resolution of these data to 100‐year time steps, by simple linear interpolation.

*Ranunculus kuepferi* is a grassland species. We hence modelled a spatially explicit time series of treeline fluctuations across the entire study area to produce a temporarily dynamic mask of forest sites unsuitable to *R. kuepferi*. The reconstruction was based on the assumption that the (potential) treeline position is changing with climatic conditions and additionally accounted for the gradual re‐forestation of the Alps during the first millennia of the simulation period and for the land use‐driven lowering of the treeline during the last millennia (see Appendix S1 for details).

### Modelling framework

We simulated the range dynamics of *R. kuepferi* using the modelling framework CATS (Dullinger *et al*. 2012; Hülber *et al*. 2016). CATS simulates the annual change in species’ occupancy and abundance at each site of a study area. Local populations are represented by stage‐structured cohorts (seeds, juveniles and adults), with annual dynamics determined by processes of stasis and transition within and among these cohorts (seed yield, seed banking, germination, juvenile survival, maturation, fecundity; here summarised as demographic rates). The demographic rates, and hence the annual seed yield as their joint outcome, differ at each site and year of simulation according to: (1) climatic/environmental suitability as predicted by a species distribution model (SDM); and (2) the density (= number of individuals) of the local population. Under constant environmental conditions, minor fluctuations of species ranges arise from stochastic events implemented in demographic and dispersal simulation routines. Under changing conditions, range dynamics result from the impact of changing environmental suitability, that is, altered SDM predictions, on the local demographic rates and the subsequent growth or decline of populations, eventual extinction from no longer suitable sites or dispersal‐mediated establishment at newly suitable sites.

### Parameterization data – SDMs, demography, dispersal

The SDMs used to project changes in the environmental suitability of individual sites for each cytotype were calibrated and validated with empirical data representing their current distribution. These data were sampled in 2013 and 2014 and contain 102 plots of 100 × 100 m^2^ spread across the entire Alpine range of the species (cf. Kirchheimer *et al*. 2016). Twenty‐three of these populations were purely diploid, 60 purely tetraploid and 19, all from a narrow contact zone in France, contained individuals of both cytotypes. We coupled these presence data with field absences (i.e. confirmed true absences for the species *R. kuepferi* sensu lato, and hence also for both cytotypes) at 8239 non‐forest plots extracted from the Alps vegetation database (Lenoir *et al*. 2012). These plots were also distributed across the entire Alps and cover elevations between 1000 and 3400 m a.s.l., that is, they bracket the elevational range of *R. kuepferi*. The combined data set was then used to parameterize four different algorithms: generalised linear models (GLM); generalised additive models (GAM); boosted regression trees (GBM); and random forests (RF). Parameterization was performed separately for the diploid and the tetraploid cytotypes as well as for the merged occurrences of both cytotypes (i.e. for *R. kuepferi* s.l.). The four algorithms used provide high to very high discrimination ability with presence–absence data and are known to be computationally efficient (e.g. Elith *et al*. 2006). For all models, we used the same set of seven predictor variables: three bioclimatic variables derived from monthly temperature and precipitation values (maximum temperature of the warmest month; annual temperature range = maximum temperature of the warmest month minus minimum temperature of the coldest month; and precipitation of the driest month); one soil variable (percentage of calcareous bedrock material); one variable indicating site‐specific incoming solar radiation; and two variables describing terrain topography (slope inclination and curvature, see Appendix S1 for details). Models were validated by calculating the area under the receiver operating curve (AUC), based on a 10‐fold cross‐validation (Van Houwelingen & Le Cessie 1990) on the calibration data set. Weighted means of occurrence probabilities projected by the four modelling techniques (‘ensemble projections’) were then used as a measure of each 100 × 100 m² site's suitability for each cytotype as well as for *R. kuepferi* s.l. (i.e. the two cytotypes in combination). Weights were determined by the AUC scores of the individual modelling techniques (see Table S2). After computing the same three bioclimatic variables for the historical climatic time series, ensemble projections were produced for each 100 years’ time slice provided by the reconstructed climate maps. For the years in between, site suitability was linearly interpolated. The result hence was an annual time series of 10 000 suitability maps per cytotype as well as for *R. kuepferi* s.l.

### Demographic modelling and pollinator competition

The suitability of a site in a particular year was translated into the cytotype's local population dynamics by relating demographic rates as sigmoidal functions to SDM projections. Demographic rates were thereby constrained between zero and a maximum value extracted from field data (or, in some cases, estimated, Table S1). In addition, germination and juvenile survival were modelled as density dependent, that is, their rates decline with the growth of the local adult population (Appendix S1).

The annual seed yield of a population at a certain site (and year) was calculated as the product of the population size and two demographic rates: flowering frequency and seed number per individual. For the sexually reproducing, diploid and outcrossing cytotype, at least two adult individuals were required for seed set. In case of mixed diploid and tetraploid populations, we moreover introduced a mechanism to represent the effect of pollinator competition on the diploids, that is, we computed the probability of pollen transfer from other diploid individuals. We thereby assumed a pollinating insect to consecutively visit 5–10 *R. kuepferi* individuals (exact number chosen randomly within that range at each site) and calculated the probability that these 5–10 visits included at least two diploid individuals from a binomial distribution with parameter *p* (the probability of a diploid individual per single visit) equal to the proportion of diploids in the entire (mixed) population of the site. The potential seed yield of the site's diploid population was then multiplied by *p* to reduce the seed yield in response to the encroachment of tetraploids. We did not implement a similar cross‐pollination effect on the apomictic tetraploids. In fact, tetraploids need pollen for endosperm fertilisation (‘pseudogamy’). However, they can use self‐pollen for this purpose (Cosendai *et al*. 2013).

### Seed dispersal

Wind is assumed to be the main dispersal vector of *R. kuepferi* diaspores (Müller‐Schneider 1986). However, as most species are likely polychorous (Nathan *et al*. 2008), and as especially large herbivores transport the seeds of many herbs (e.g. Vellend *et al*. 2003), we combined dispersal functions for four different processes likely to transport diaspores over longer distances: by wind, within the furs and guts of large herbivores, respectively, and by an unspecified long‐distance dispersal (LDD) vector. Wind dispersal was modelled by means of the analytical WALD kernel (Katul *et al*. 2005). Exo‐ and endozoochorous kernels were parameterized on the basis of correlated random walk simulations for the most frequent large herbivore in the study area, the chamois (*Rupicapra rupicapra* L.). Rare LDD events were accounted for by distributing 0.1% of a population's seed yield randomly within a radius of 50 km. For details on seed dispersal functions, see Appendix S1.

### Simulation set‐up

To identify the relative effects of niche differentiation and mode of reproduction (plus their possible interaction effect) on the Holocene range development of the two cytotypes, we ran a factorial design of simulations with two variables: niche (two levels: own niche vs. common niche of *R. kuepferi* s.l.) and reproductive mode (three levels: own mode – both sexual – both asexual). To account for (pollinator) competition among cytotypes, simulations were run simultaneously for both cytotypes. For each factorial combination, three replicate simulations were run. We refrained from a larger number of replicates as results did not vary substantially among replicates and computing efforts per replicate were extremely high.

**Figure 2:**
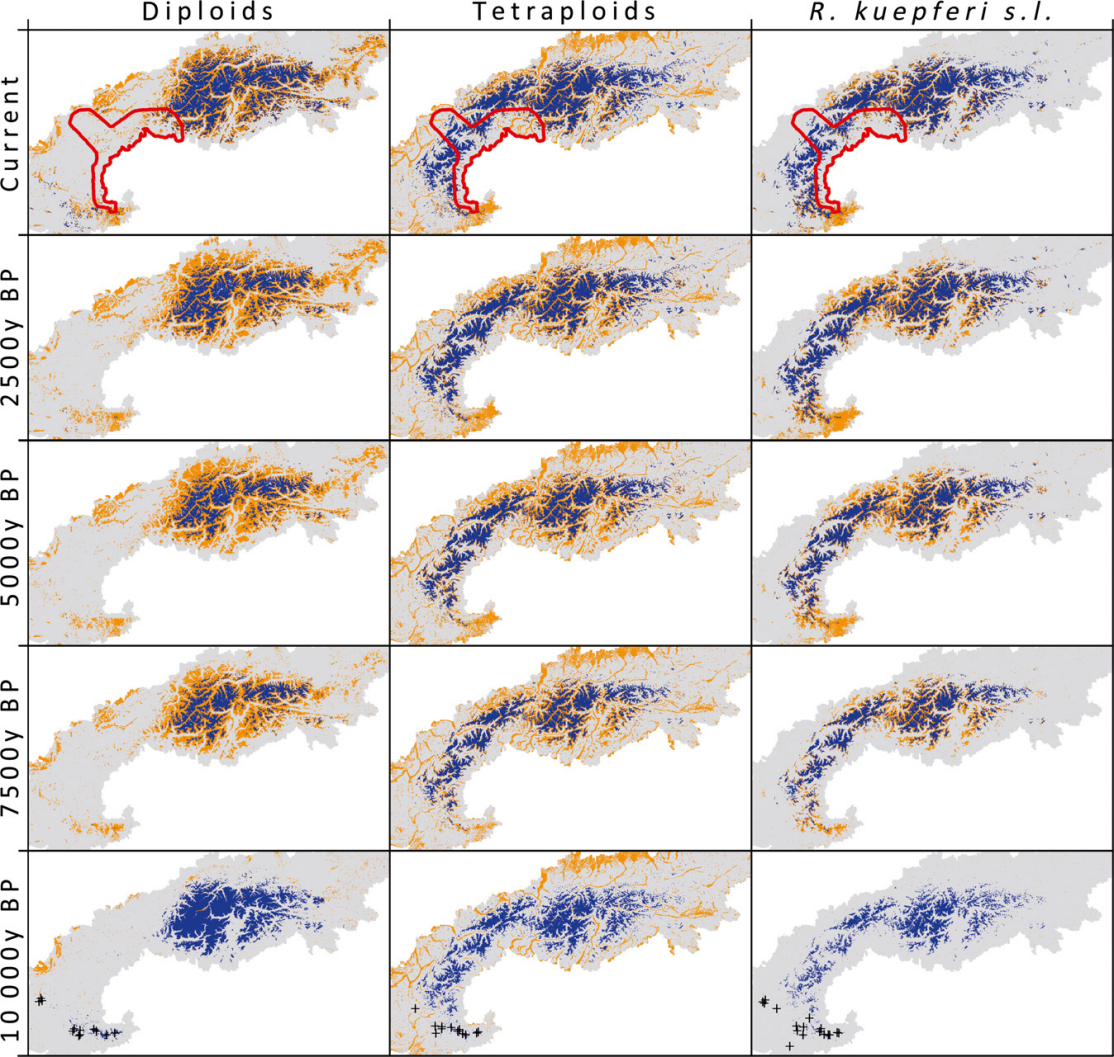
Potential ranges of the two cytotypes of *Ranunculus kuepferi*, and of *R. kuepferi* s.l. in the European Alps (grey) at five different times. Coloured sites are those which are climatically suitable and either above (blue) or below (brown) the modelled treeline at the respective time. Crosses mark the geographical positions of the initial populations, that is, those sites assumed to be occupied at the start of the simulations (10 kyr BP). Red polygons in the ‘current’ row represent the main break zone in Alpine species distributions identified in Thiel‐Egenter *et al*. (2011). To improve visibility, maps have been resampled to a resolution of 1 × 1 km² with all cells containing at least one suitable 100 × 100 m² site coloured appropriately. See Fig. S2 for an original resolution map.

The initial distribution of each of the two cytotypes, that is, the sites set as occupied in the first year of simulation (10 kyr BP), was restricted to the regions assumed to comprise the glacial refugia of the species in the south‐western Alps (Burnier *et al*. 2009). We identified regions of particular high suitability at 10 kyr BP within a radius of 10 km around each documented population (12 diploid and 12 tetraploid populations, Fig. 2). Each site within these regions was assumed to be inhabited by a population of half of the site's carrying capacity. We started simulations in 10 kyr BP as the available climatic reconstructions are limited to this period. However, in most areas of the Alps, the main deglaciation did not take place before the end of the Younger Dryas, that is, between 11.9 and 10.5 kyr BP (Darnault *et al*. 2012). Even if distribution ranges of both cytotypes in the south‐western Alps may already have been larger than we assumed, it is hence highly unlikely that the tetraploid cytotype had emerged and moved out of the most south‐western Alps before 10 kyr BP.

### Statistical analyses of the simulations’ outputs

From the final result of each simulation run, that is, the cytotype distributions reached in the last simulation year, we calculated the convex hull around the diploid and tetraploid distribution ranges respectively. The convex hull is the smallest convex area that contains all sites where the respective cytotype is present and was calculated using the *convexhull* function in the R‐package ‘siar’ (version 4.2, Parnell *et al*. 2013). Areas outside the Alpine chain were removed from the resulting hulls.

For each simulation run, we then divided the resulting convex hull area of the tetraploids by the convex hull area of the diploids. The log of this ratio was subtracted from the log of the same ratio applied to the actual distribution of the two cytotypes. This index hence measures how precisely the simulations recapture the observed range size ratio of the two cytotypes. We subsequently used this metric as the response variable in a linear regression with niche, reproductive mode and their interaction as predictors. The variance explained by these factors was computed from their respective partial *R* ^2^ values. As a backup, we additionally calculated and inspected ΔAIC_c_ values of the candidate models using the *aictab* function of the R‐package ‘AICmodAVG’ (version 2.1‐1, Mazerolle 2013).

## Results

### Species distribution models

Evaluation scores of SDMs indicate reasonable model projections for both cytotypes as well as for *R. kuepferi* s.l. (cross‐validated AUC 0.76–0.85, see Table S2). Niche differences among the two cytotypes (Kirchheimer *et al*. 2016) translate into different predictions of potential geographical ranges (compare blue pixels in Fig. 2 and Fig. S2). In particular, the preference for cooler temperatures shifts the potential range of the tetraploid to higher elevations (mean elevation of diploids: 2367 ± 279 m a.s.l.; tetraploids: 2527 ± 349 m a.s.l.). Across the last 10 kyr, potential ranges fluctuate for both cytotypes (Fig. 2). Initially (10 kyr BP), the range size is about equal for both cytotypes (*c*. 1200 km²) and decreases markedly towards 5 kyr BP, in particular for diploids (*c*. 600 km). During the last millenia, the range sharply increases again for diploids (*c*. 1500 km²), but further decreases for tetraploids (*c*. 700 km²). These dynamics are partly driven by the relatively modest climatic changes during the last 10 kyr BP (Figs S3 and S4). More importantly, potential ranges overlap to different degrees with forest cover during the last 10 kyr. This overlap is stronger for the diploids, which prefer warmer conditions and hence have a higher share of suitable habitats below the climatic treeline (compare brown pixels in Fig. 2). As a consequence, their potential range shrinks sharply with the rise of treeline during the first two millennia, but increases again with the human‐driven lowering of treeline during the most recent millennia.

Apart from differences in size and its fluctuations over time, the geography of potential ranges also differs between the two cytotypes. While suitable sites concentrate in the central parts of the Alps for both cytotypes, the density of suitable sites in large parts of the western Alps is much lower for diploids than for tetraploids (Fig. 2). Hence, for diploids, putative ice age refugia and current areas with high environmental suitability are separated by a pronounced gap, whereas tetraploids had a higher density of stepping stones between glacial refugia and current ‘hotspots’ of suitable areas.

Surprisingly, at first glance, the modelled niche of *R. kuepferi* s.l. is narrower than the one of the two cytotypes. However, adding together two occurrence probability distributions (along an environmental gradient) results in a combined distribution where the overlapping part is emphasised (because occurrence probabilities of both cytotypes add together), while those parts ‘private’ to only one cytotype are down‐weighted (in relative terms; because only one cytotype contributes its occurrence probability). Combining niches of two taxa in probabilistic SDMs hence accentuates the common niche space and can thus easily predict a weighted niche that is narrower than the one(s) of the two constituent taxa. In the case of *R. kuepferi*, this resulted in a projected potential range of *R. kuepferi* s.l. smaller than those of the individual cytotypes or intermediate between them (Fig. 2). The geographical distribution of these suitable sites, however, is closer to those of the tetraploids, likely because tetraploids are more frequent (and hence had more weight) in the data set used for parameterising the SDMs.

### Simulation of range dynamics

**Figure 3:**
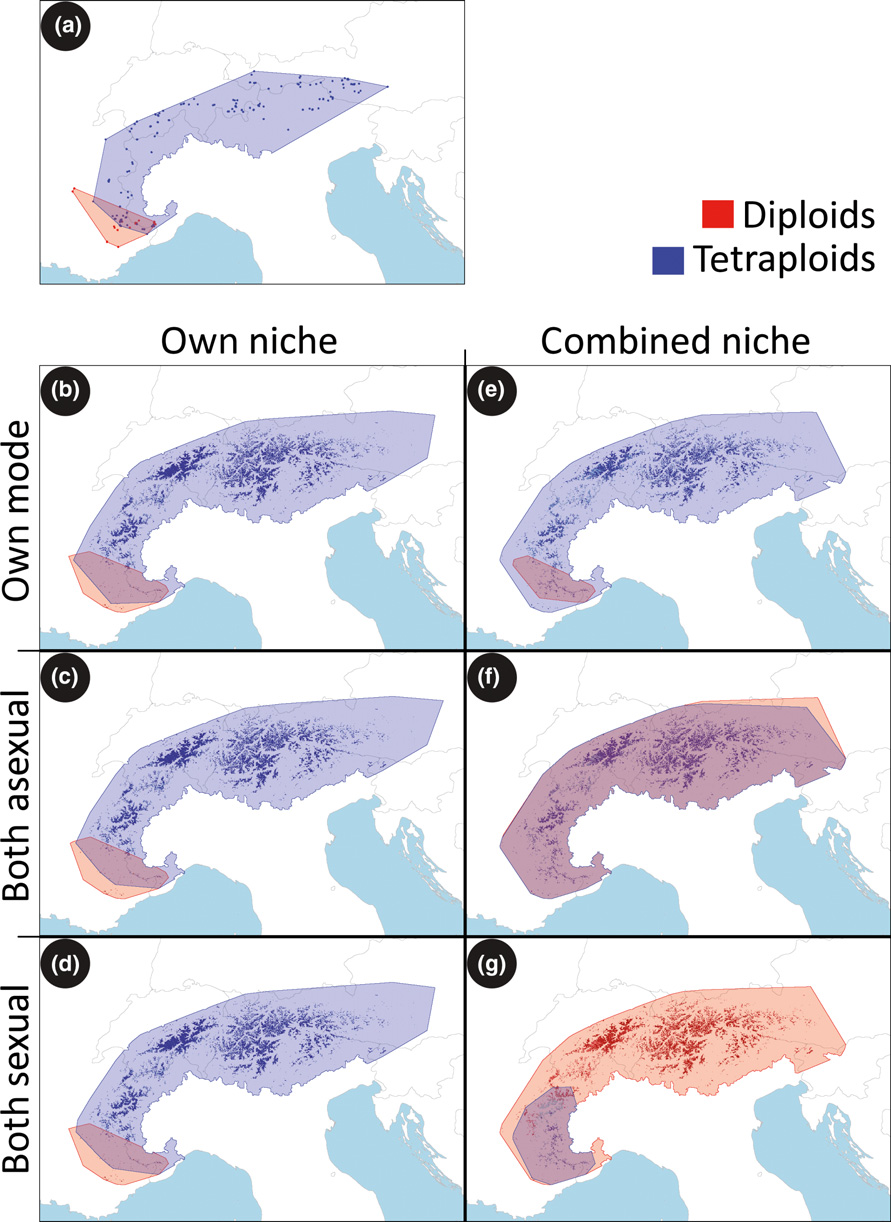
Convex hull areas around the observed current distribution of the two cytotypes of *Ranunculus kuepferi* (a) and around the current distributions simulated under the assumption that cytotypes have their specific climatic niches (b–d) or the merged niche of both cytotypes (e–g); and that they have their specific reproduction mode (b, e) or that both cytotypes are either apomicts (c, f) or sexual outcrossers (d, g). Each panel represents the result of a randomly selected replicate from the respective simulation setting (see Figs S6 and S7 for the results of the other two replicates). Background shading indicates sites (= raster cells) occupied by the species at the end of the simulation period. Areas outside the Alpine chain were removed from the hulls.

**Figure 4:**
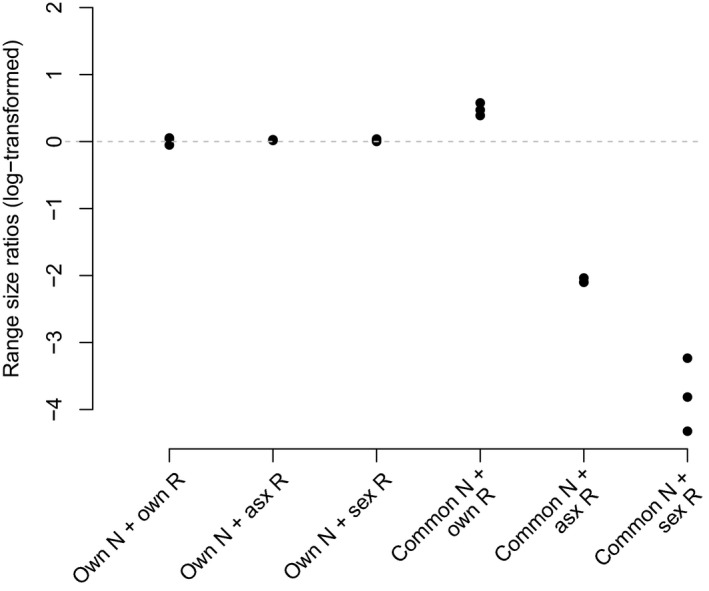
Convex hull area ratios of the two cytotypes resulting from simulations under the 2 × 3 factorial combinations of the cytotypes’ niche (own N vs. common N of *R. kuepferi* s.l.) and reproductive modes (own R – sexual R – asexual R). Each dot represents one replicate simulation run. Values were calculated by dividing the convex hull area of the tetraploids by the convex hull area of the diploids. The log of this ratio was subtracted from the log of the same ratio applied to the real ranges of the two cytotypes. A value of 0 implies that simulated and real ratios do not differ.

When running the simulation models with each cytotype's own niche and reproductive mode, simulations slightly overestimate the empirical ranges of both cytotypes, but recapture the ratio of the two ranges quite accurately (Figs 3a,b and 4). Accounting for niche differentiation among cytotypes, but assuming that both cytotypes had the same reproductive mode, does not change these results (Figs 3c,d and 4). Independent of the reproductive mode, the cytotype with the ‘tetraploid niche’ successfully spreads across much of the Alps, while the cytotype with the ‘diploid niche’ remains restricted to the south‐western refugia.

Nevertheless, reproductive modes are relevant for simulation results. When the two cytotypes are assumed to share a common niche of *R. kuepferi* s.l. (Fig. 2) but to keep their own reproductive modes, simulations overemphasise observed range differences. While tetraploids recapture their currently realised range, diploids are confined to an area even smaller (Fig. 3e). Additional simulations where both cytotypes share the tetraploid niche (instead of the one of *R. kuepferi* s.l.) delivered very similar results (Fig. S5b). When both cytotypes are assigned the diploid niche, the asexually reproducing one remains restricted to the south‐western Alps, while the sexually reproducing one goes extinct (Fig. S5a). Taken together, simulations that neglect niche differentiation, but account for differences in the reproductive mode produce biased observed range size ratios that favour the asexual cytotype.

Finally, assigning both cytotypes the same niche (of *R. kuepferi* s.l.) and reproductive mode delivered contrasting results. If both cytotypes are assumed to reproduce asexually, they both spread across the Alps (Fig. 3f) and colonise approximately equal ranges. If both cytotypes are prescribed to reproduce sexually, however, the diploid expands across the Alps, while the tetraploid gets stuck in the western Alps (Fig. 3g). Because, in this setup, the two cytotypes are assumed to be identical with respect to niche and reproductive mode, and demographic and dispersal parameters are very similar (Table S1), this result suggests that sexual reproducers are potentially much more sensitive to priority effects and competition for range formation.

Variance partitioning and the AIC‐based evaluation summarise these results: they consistently suggest that both niche differentiation and differences in reproductive modes have important and independent effects on simulation outcomes, with niche differentiation being slightly more influential (Table 1). Moreover, they highlight the importance of the interaction effect among the two factors which explains about half of the total variance of the simulation results.

**Table 1:** Adjusted *R* ^2^ and AIC_c_ values of linear regression models explaining simulation results by the two factors explored (Reproduction with levels own – sexual – asexual; Niche with levels own – common niche of *R. kuepferi* s.l.), and their interaction respectively. The response variable was the difference between simulated and observed (log‐transformed) range size ratios of the two cytotypes as presented in Fig. 3. AIC_c_ is the Akaike Information Criterion corrected for small sample size, calculated following Burnham & Anderson (2002)

Terms	Adj. *R*²	AICc
Reproduction	0.23	71.0
Niche	0.30	66.9
Reproduction + Niche	0.59	46.5
Reproduction × Niche	0.98	15.7

## Discussion

Taken together, our results underline the complex causation of geographical parthenogenesis (Hörandl 2006). Both niche differentiation and change in reproductive mode can have significant independent and strong interacting consequences for the development of ranges in sexual and asexual *R. kuepferi*. The observed range size difference is apparently determined primarily by niche differentiation, as assigning the cytotypes their own niches reproduces current range size ratios and range positions quite accurately, independent of assumed reproductive modes. This niche differentiation opened migration pathways to the more cold‐adapted tetraploid cytotype which were not available to the diploid cytotype (compare blue pixels in Fig. 2). Eastward expansion of the latter was impossible even if simulations assumed that it would be able to reproduce uniparentally. However, niche differentiation is not a generic prerequisite (or consequence) of the emergence of (polyploid) apomicts in plants (Glennon *et al*. 2014). Assuming that such differentiation did not happen, as in our simulation design (Fig. 3e,f,g), is hence likely appropriate for other cases of geographical parthenogenesis. For such cases, our results suggest a potentially strong effect of the separation of reproductive modes on the cytotype's range sizes: under assumed identical reproductive modes (and the identical niche of *R. kuepferi* s.l.) either both cytotypes or only the sexual one was able to expand across the Alps. By contrast, implementing a switch to apomixis on the side of the tetraploids turns simulation results around and even overemphasizes the superiority of the tetraploid asexual cytotype (Fig. 3g).

### Geographical parthenogenesis in *R. kuepferi*

The dominant effect of niche differentiation on the current geographical parthenogenesis in *R. kuepferi* is surprising at first glance because the tetraploid's niche is not broader than the one of the diploid (Kirchheimer *et al*. 2016) and its potential current range is smaller. Rather than niche breadth, differences in niche position were hence responsible for the emergence of vastly different ranges. In particular, tetraploids’ preference for colder conditions caused the post‐glacial re‐encroachment of forests into the Alps to interfere more with the area suitable for diploids than for tetraploids. In addition, the unique topography of the Alps was apparently crucial. The western Alps represent an area of pronounced topographical complexity owing to particularly high elevations interspersed with deep glacial valleys. This complexity constituted an efficient barrier to the post‐glacial recolonisation of the Alps from marginal refugia for many alpine species (Merxmüller 1952). As a consequence, the area currently represents the most prominent ‘break zone’ in the distribution of both species and alleles in the European Alps (Thiel‐Egenter *et al*. 2011; Fig. 2). The tetraploid cytotype of *R. kuepferi* has a higher density of suitable sites in this area because its niche expands further into the regionally prevailing cool and humid conditions (Kirchheimer *et al*. 2016). By contrast, nearly all sites climatically suitable to diploids are below treeline, which is particularly high in this region due to pronounced climatic continentality (Ozenda 1985).

Our results do not support a primary role of faster migration ability (Baker's Law) in generating geographical parthenogenesis in *R. kuepferi*. Sexual outcrossers were simulated to spread across the entire Alps as did apomicts with the ‘appropriate’ niche. This does not, of course, mean that spread rates are independent of the reproductive mode, but that the past 10 kyr were long enough to allow even outcrossing *R. kuepferi* to recolonise most parts of the Alps if suitable stepping stones had been available. Indeed, many widespread alpine species are outcrossers (Schroeter 1908; Körner 2003), even at the highest elevations (Hörandl 2011). Sexual plants hence obviously managed a sufficiently rapid range expansion throughout the Alps despite being dependent on the co‐occurrence of mating partners.

### Geographical parthenogenesis beyond *R. kuepferi*

A characteristic feature of geographical parthenogenesis is that ranges of asexual taxa are not only larger but also often extend further into cold environments that have been glaciated during the Pleistocene (Bierzychudek 1985). Preference, or at least tolerance of cooler conditions by apomicts appears hence frequently linked to this biogeographic phenomenon. In apomict plants, this shift is likely mediated by bypassing meiosis and becoming independent of pollinators, two features that confer advantages in cold climates. However, this shift has so far primarily been reported as evidence of niche broadening that subsequently enabled range expansion (Hörandl 2006). In contrast, our results for *R. kuepferi* suggest that a preference for cooler conditions *per se* may have been responsible for greater distributional success of asexual lineages. As geographical parthenogenesis is particularly frequent in alpine floras (Asker & Jerling 1992; Hörandl 2011) *R. kuepferi* is unlikely a special case. For all these non‐forest species, adaptations to cool conditions have increased the permeability of mountain landscapes during the Holocene because they helped these species escape the rising treeline and migrate across higher mountain ranges. Additional support for this assumption comes from the biogeography of narrow range endemics: among vascular plants of the Austrian Alps, such endemics are most frequent at subalpine elevations and decline rapidly in the mid and upper alpine belts (Essl *et al*. 2009). Post‐glacial recolonisation of the Alps from marginal refugia was hence easier for species adapted to high than to intermediate elevations.

Our simulations also suggest, however, that identifying any primary mechanism responsible for geographical parthenogenesis is difficult because subtle differences can completely reverse biogeographic patterns. The fundamentally different results illustrated in Fig. 3f and g are particularly instructive in this respect and illustrate the potential importance that differentiation of reproductive modes can have for the emergence of geographical parthenogenesis. In both cases, the two cytotypes are assumed to have identical reproduction modes and a niche that allows colonisation of the full species’ range (= the niche of *R. kuepferi* s.l.). They only differ in the assumed position of initial populations in the south‐western Alps at the simulation start in 10 kyr BP. These minor differences are irrelevant as long as both cytotypes are assumed to reproduce asexually, but result in major range size differences if they are assumed to be sexual outcrossers. In this latter case, the sensitivity of sexually reproducing taxa to minority cytotype disadvantage amplifies priority effects: differences in positions of initial populations give one cytotype a head start. The other cytotype has to migrate through ‘occupied terrain’ which greatly delays its own spread (see Appendix S2). For any real‐world case of geographical parthenogenesis, the implication is (1) that a switch to apomixis immunises the asexual cytotype against the disadvantage of necessarily being the minority cytotype initially, and hence any consequent priority effects; and (2) that, during phases of post‐glacial warming, the spread of sexual populations may have been blocked by populations of their relatives, especially by asexual, but also by sexual ones, resulting in eventual ranges much smaller than niche requirements would allow. In light of these results, niche differentiation may not only have fostered establishment of the derived cytotype, but may have also facilitated the survival of the progenitor because it prevented the derived cytotype from pre‐occupying all terrain that became suitable to the cytotypes (Fig. S5).

## Conclusions

Taken together, our results suggest that in *R. kuepferi,* and probably in other taxa of temperate mountain ranges, the distributional advantage of asexuality is determined by a complex interaction between plant species and their abiotic and biotic environment. Loss of sex (Bomblies *et al*. 2015; Mirzaghaderi & Hörandl 2016) and the concomitant polyploidization (Bierzychudek 1985; Brochmann *et al*. 2004; Comai 2005) here fosters tolerance of cooler conditions. Tolerance to cooler conditions then facilitates re‐immigration into (previously glaciated) mountain ranges because this tolerance helps overcoming high elevation barriers. In addition, loss of sex confers competitive advantages to asexual lineages that can modify or even reverse the distributional consequences of priority effects and enable asexual populations to block further migration of their sexual relatives. These findings do not imply that niche broadening and colonisation advantages of uniparental reproduction, the main mechanisms behind geographical parthenogenesis of plants discussed so far, are unimportant. They suggest, however, that at least in temperate mountains, niche shifts towards cooler conditions and competitive advantages of asexuality may even be more important to understand geographical parthenogenesis.

## Author Contributions

BK, JW, EH and SD designed research. BK, KH, CCFS, SK, AD, EH and SD conducted field sampling. MC, AG, JL, DNL, JCS, WW and SD contributed relevé data. MA performed age determination of the tetraploid cytotype. DM, MK and CP performed GIS work. JW and AG conducted simulations. BK analysed simulation results. BK and SD wrote the paper. All authors commented on and discussed previous versions of the paper.

## Data Accessibility Statement

Data on the distribution of *R. kuepferi* cytotypes will be submitted to DRYAD upon acceptance of the paper.
