# Supplementary material for: Reconstructing geographical parthenogenesis: effects of niche differentiation and reproductive mode on Holocene range expansion of an alpine plant
Source: Ecol Lett. 2018 Jan 19;21(3):392–401. doi: 10.1111/ele.12908 (PMC5888191; doi:10.1111/ele.12908)

**Figure S1** Dated phylogenetic tree from the BEAST analysis based on ITS, rpl20-rps12 and trnL-trnF sequence data. The statistical support for clades is shown above branches as Bayesian posterior probabilities (pp). Only pp values for supported clades are displayed­­ and unsupported clades are marked with a dash (-). Clades without statistical support of internal branches are shown as triangles. The mixed diploid and tetraploid populations from the SW Alps form a strongly supported clade (highlighted in green colour). The age estimations in millions of years ago (MA) are displayed below the branches for supported clades. The first value corresponds to the mean age followed by the credibility interval in brackets.


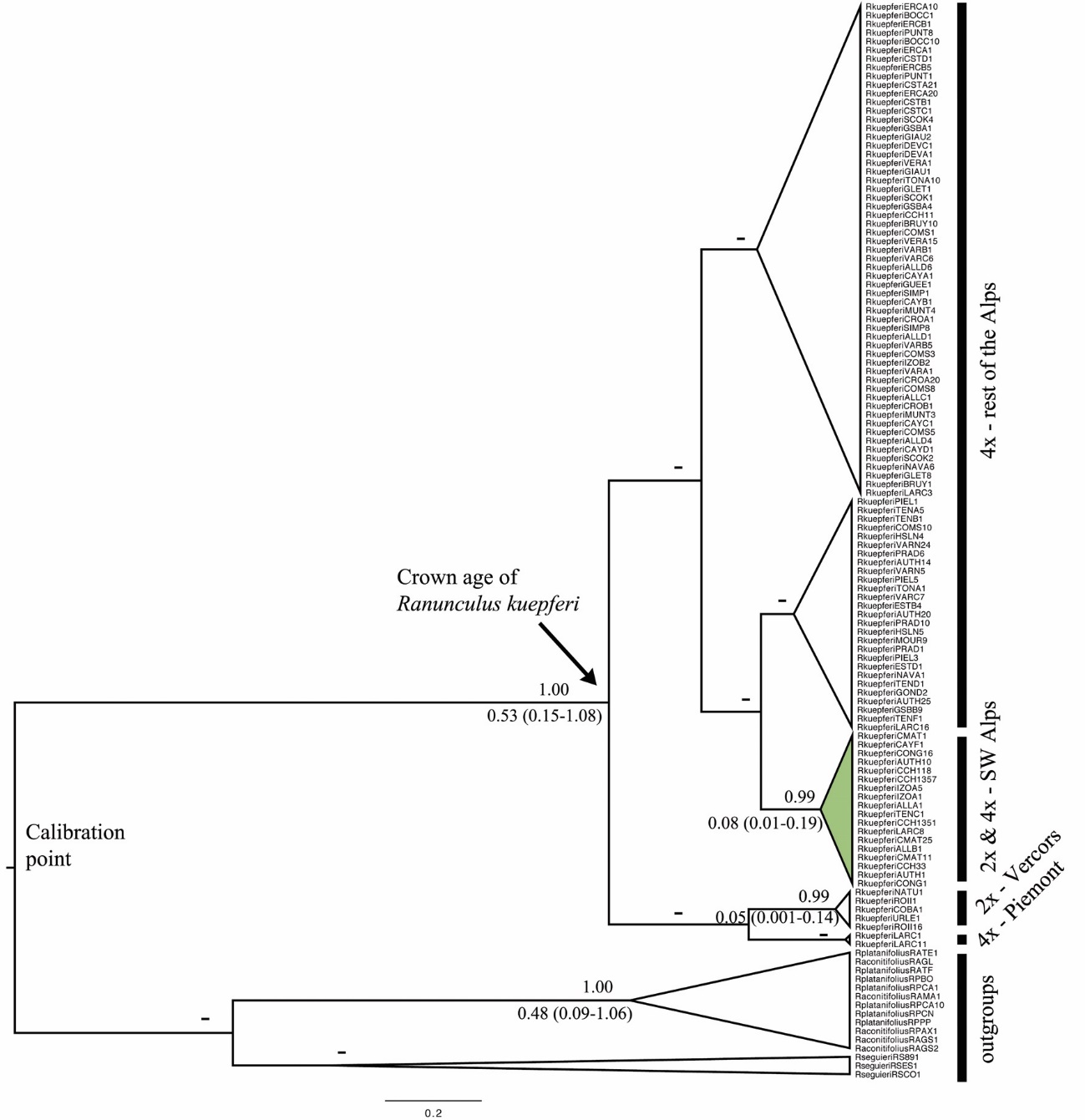

Supplement: Supplementary file 1 [file ELE-21-392-s001.docx]
