# Supplementary material for: Reconstructing geographical parthenogenesis: effects of niche differentiation and reproductive mode on Holocene range expansion of an alpine plant
Source: Ecol Lett. 2018 Jan 19;21(3):392–401. doi: 10.1111/ele.12908 (PMC5888191; doi:10.1111/ele.12908)

**Figure S2** Potential ranges of the two cytotypes of *R. kuepferi*, and of *R. kuepferi* s.l. in the European Alps (grey) at five different times. Coloured sites are those which are climatically suitable and either above (blue) or below (brown) the treeline at the respective time. Crosses mark the geographical positions of the initial populations, i.e. those sites assumed to be occupied at the start of the simulations (10 kyr BP). Polygons in the ‘current’ row represent the main break zone in Alpine species distributions identified in Thiel-Egenter et al. (2011).


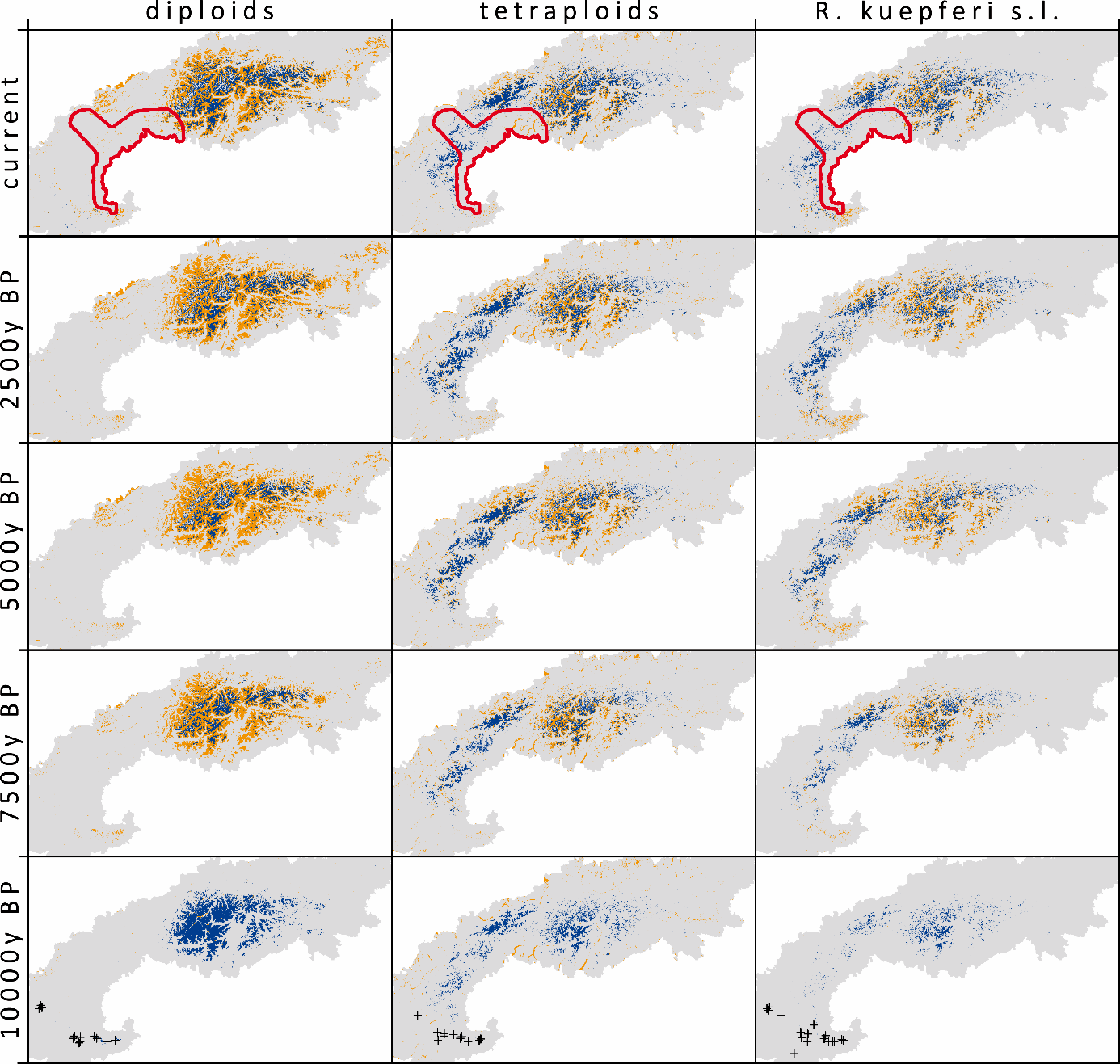

Supplement: Supplementary file 2 [file ELE-21-392-s002.docx]
