# Supplementary material for: Reconstructing geographical parthenogenesis: effects of niche differentiation and reproductive mode on Holocene range expansion of an alpine plant
Source: Ecol Lett. 2018 Jan 19;21(3):392–401. doi: 10.1111/ele.12908 (PMC5888191; doi:10.1111/ele.12908)

**Figure** S4 Changes in annual precipitation sums in the study area between 1300 and 2800 m elevation during the last 10 k years. The blue line and the blue shaded area show the average and standard deviation, respectively, across all 100 x 100 sites of the study area.


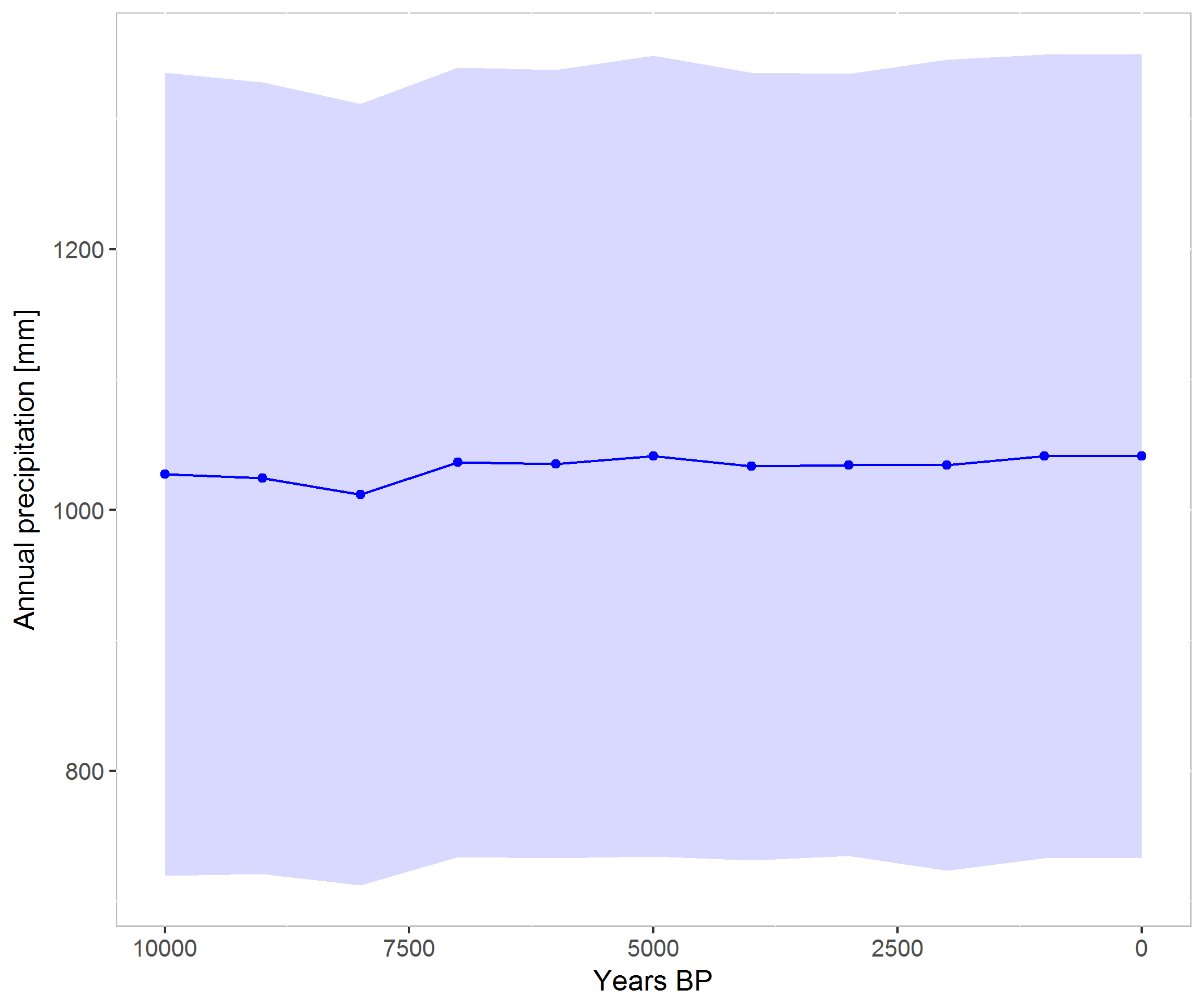

Supplement: Supplementary file 4 [file ELE-21-392-s004.docx]
