# Supplementary material for: Reconstructing geographical parthenogenesis: effects of niche differentiation and reproductive mode on Holocene range expansion of an alpine plant
Source: Ecol Lett. 2018 Jan 19;21(3):392–401. doi: 10.1111/ele.12908 (PMC5888191; doi:10.1111/ele.12908)

**Figure S5** Convex-hull areas of the *R. kuepferi* cytotype distributions. Polygons show the convex-hulls around the current distributions simulated under the assumption that both cytotypes share either the diploid’s (a) or the tetraploid’s (b) niche but have their own reproductive modes, respectively. Each panel represents the result of a randomly selected replicate from the respective simulation setting. Red polygons refer to the diploid cytotype and blue polygons to tetraploid cytotype.


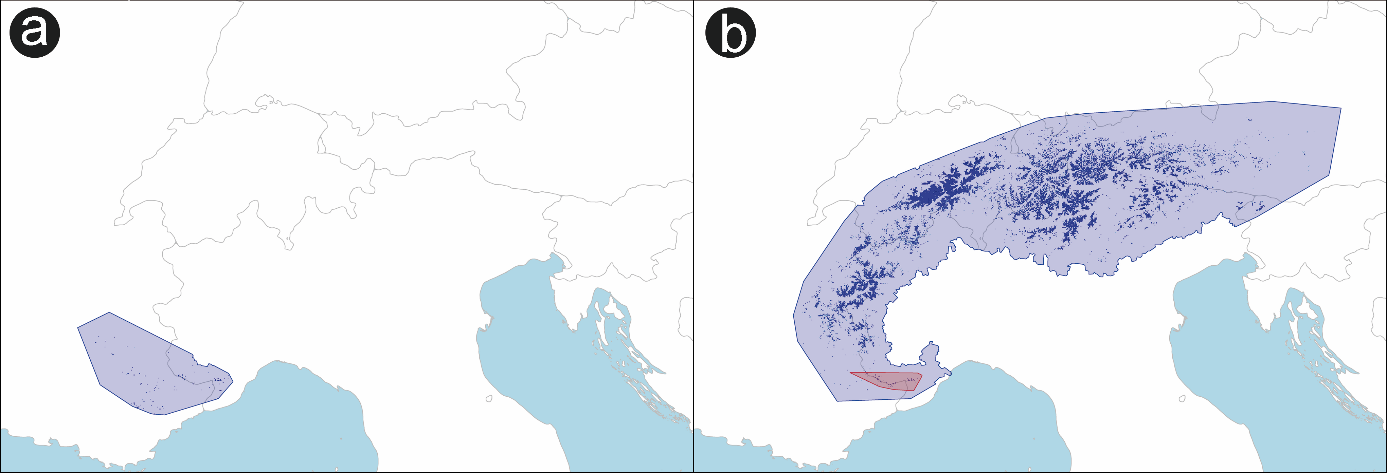

Supplement: Supplementary file 5 [file ELE-21-392-s005.docx]
