# Supplementary material for: Reconstructing geographical parthenogenesis: effects of niche differentiation and reproductive mode on Holocene range expansion of an alpine plant
Source: Ecol Lett. 2018 Jan 19;21(3):392–401. doi: 10.1111/ele.12908 (PMC5888191; doi:10.1111/ele.12908)
Supplement: Supplementary file 6 [file ELE-21-392-s006.docx]

**Figure S6** Convex-hull areas around the observed current distribution of the two cytotypes of *R. kuepferi* (a) and around the current distributions simulated under the assumption that cytotypes have their specific climatic niches (b-d) or the merged niche of both cytotypes (e-g); and that they have their specific reproduction mode (b, e) or that both cytotypes are either apomicts (c, f) or sexual outcrossers (d, g). The panels represent the result of the second replicate from the respective simulation settings. Background shading indicates sites (= raster cells) occupied by the species at the end of the simulation period. Areas outside the Alpine chain were removed from the hulls.

**
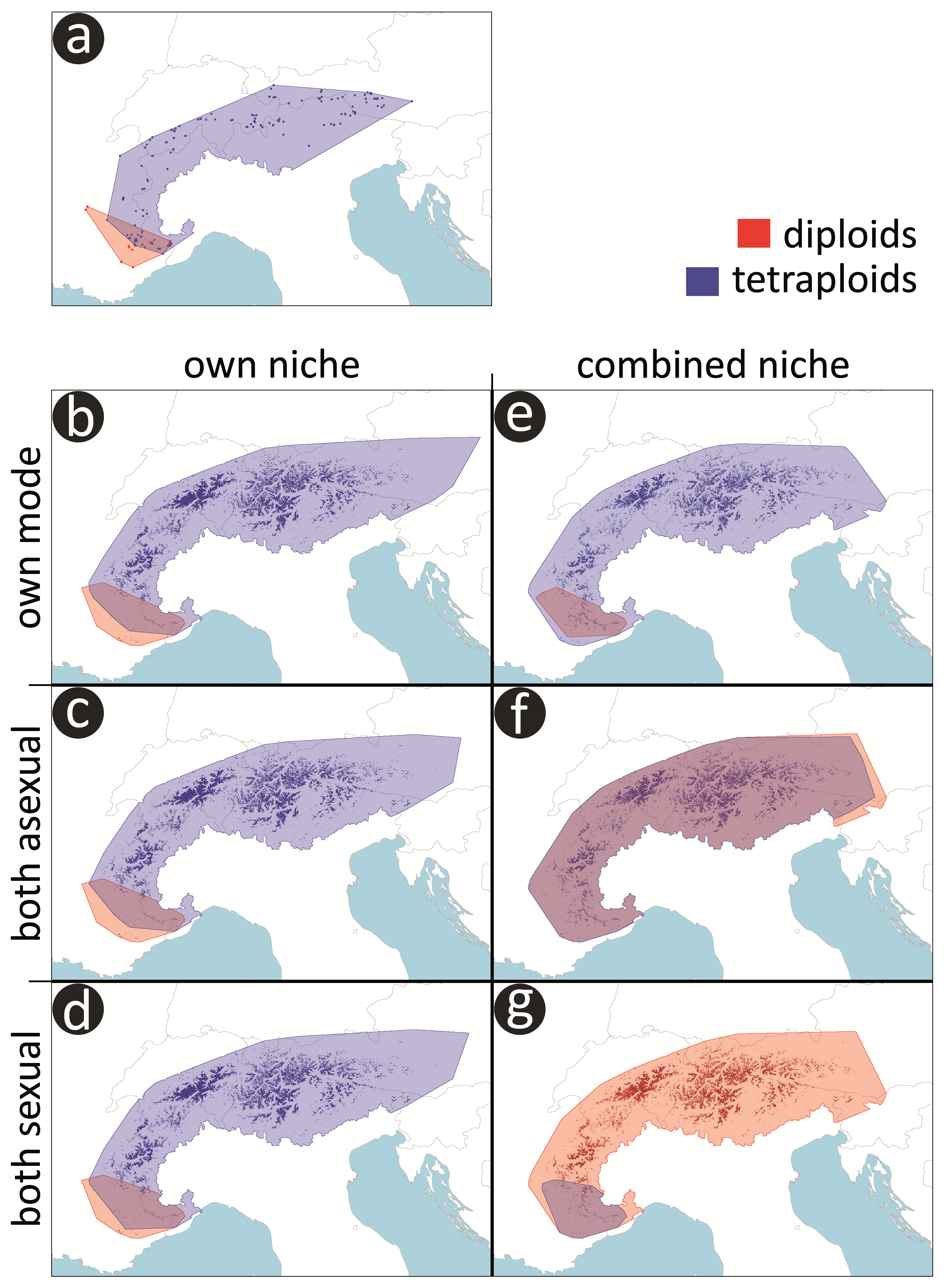
**
