# Supplementary material for: Reconstructing geographical parthenogenesis: effects of niche differentiation and reproductive mode on Holocene range expansion of an alpine plant
Source: Ecol Lett. 2018 Jan 19;21(3):392–401. doi: 10.1111/ele.12908 (PMC5888191; doi:10.1111/ele.12908)
Supplement: Supplementary file 8 [file ELE-21-392-s008.docx]

**Table S1** Maximum demographic rates for the diploid and tetraploid cytotype and *R. kuepferi* s. l., and their traits relevant for constructing dispersal kernels.

| **Species** | **diploid** | **tetraploid** | ***R.k.s.l.*** |
| --- | --- | --- | --- |
| **Mat** | 4 – 7 | 4 – 7 | 4 – 7 |
| **Age** | 27 | 27 | 27 |
| **SP_H** | 10 | 10 | 10 |
| **SP_L** | 5 | 5 | 5 |
| **CC_H** | 1.2 * 10^6^ | 1.3 * 10^6^ | 1.6 * 10^6^ |
| **CC_L** | 3.2 * 10^5^ | 1.5 * 10^5^ | 1.9 * 10^5^ |
| **FF_H** | 0.48 | 0.48 | 0.49 |
| **FF_L** | 0.37 | 0.39 | 0.40 |
| **JS_H** | 0.93 | 0.92 | 0.92 |
| **JS_L** | 0.46 | 0.46 | 0.46 |
| **adult_trans_max** | 0.3 | 0.3 | 0.3 |
| **adult_trans_max** | 0.15 | 0.15 | 0.15 |
| **SY_H** | 29 | 30 | 30 |
| **SY_L** | 22 | 22 | 22 |
| **Germ_H** | 0.382 | 0.390 | 0.389 |
| **Germ_L** | 0.286 | 0.292 | 0.292 |
| **H_0_** | 0.137 | 0.137 | 0.137 |
| **h** | 0.035 | 0.034 | 0.034 |
| **Diaspore mass** | 1.091 | 1.495 | 1.311 |
| **Vs** | 0.023 | 0.035 | 0.029 |
| **vt** | 2.8 | 3.2 | 3 |
| **GS** | 0.015 | 0.012 | 0.013 |
| **DR** | 37.195 | 33.455 | 35 |

**Mat** – Age of maturity (minimum – maximum years); own estimates according to cultivation of our seedlings (see Germ_H and Germ_L). The species is clearly perennial, as it is typical for apomictic plants (Asker and Jerling 1992, Richards 1997).

**Age** – Maximum age of genets (years); in cultivation, adult plants collected in 2013 survived until 2017 without any signs of senescence. As there is no detailed information on the lifespan of *R. kuepferi* available in literature, we applied the maximum age of genets reported from *Ranunculus glacialis* (Jarvinen 1989).

**SP_H and SP_L** – High and low persistence of seeds in the soil seed bank; the classification (10 long-term persistent, 5 short-term persistent,1 transient) was based on the achene shape following (Thompson *et al.* 1993). The achene shape variance was calculated from our own measurements on 68 seeds from 9 diploid populations and 81 seeds from 11 tetraploid populations. For this purpose we measured the length, breath and height with the accuracy of 10^-6^ m using a binocular microscope.

**CC_H and CC_L –** High and low carrying capacity (number of individuals or shoots); own measurements (CC_H: mean of the 5 largest populations; CC_L: mean of all populations respectively).

**FF_H and FF_L** – High and low flowering frequency (proportion of individuals/shoots flowering per year). Own measurements: we counted all flowering and not flowering individuals within an area of four 0.5 x 0.5 m in the 102 sampled populations (3^rd^ quartile (FF_H) and median (FF_L) of plot means).

**JS_H and JS_L** – High and low juvenile survival (proportion of juveniles surviving from one year to the next); because of the limited time for this study JS_H was calculated as percentage of seedlings which survived the first winter (see description of Germ_H and Germ_L). (JS_L = half of observed JS_H).

**SY_H and SY_L** – High and low seed yield (estimated from total number of well-developed achenes produced per flowering individual/shoot); the number of seeds produced per flowering individual/shoot was calculated from collected individuals (cf. Kirchheimer *et al.* 2016) (SY_H: 3^rd^ quantile; SY_L: median).

**Germ_H and Germ_L** – High and low germination rates; Germ_H was calculated as percentage of germinating seeds which survived the first winter (2014/2015). Achenes were sown in October 2013 and cultivated outdoor in Clausthal (Harz, Germany) for the first winter. By the end of May 2014 the seeds were transported to the botanical garden of the University of Göttingen were they were also cultivated outdoor. We used 110 seeds from diploid plants and 970 seeds from tetraploid plants. (Germ_L = Germ_H*0.75).

**H_0_** – Diaspore release height (m); calculated from our sampled individuals in the field using the distance from the base of the flowering sprout to the base of bud, flower or fruiting head.

**h** – Mean height of the vegetation surrounding a fruiting plant (m); measured at a distance of 0.1 m from our sampled individuals for the four cardinal directions.

**Diaspore mass** – (mg); means of own measurements of 68 achenes from 9 diploid populations and 81 achenes from 11 tetraploid populations (accuracy of 10^-3^ g).

**Vs** – Diaspore shape variance (Thompson *et al.* 1993); own measurements.

**vt** – Terminal falling velocity (m/s); taken from *Ranunculus* species which have similar looking diaspores and similar diaspore weight (for the diploids from *R. acris* from the LEDA Traitbase (Kleyer *et al.* 2008), for the tetraploids from *R. villarsii* from ECOCHANGE (own measurements taken from Dullinger *et al.* 2012) and the mean of this two values for *R. kuepferi* s. l..

**GS** – Probability to survive the gut passage of a large mammal (fallow deer) as calculated from the achene mass by the regression equation in (Moussie 2004).

**DR** – Hourly detachment rate (%) of achenes from chamois fur; assumed to be equal to the mean of the detachment rates from sheep and cattle fur as calculated from the achene mass and surface structure by the regression equations in (Römermann *et al.* 2005). Achene surface structure was classified from own samples of achenes.
