# Supplementary material for: Reconstructing geographical parthenogenesis: effects of niche differentiation and reproductive mode on Holocene range expansion of an alpine plant
Source: Ecol Lett. 2018 Jan 19;21(3):392–401. doi: 10.1111/ele.12908 (PMC5888191; doi:10.1111/ele.12908)
Supplement: Supplementary file 9 [file ELE-21-392-s009.docx]

**Table S2** AUC scores of species distribution models fitted for the two cytotypes separately, and in combination (*R. kuepferi* s.l.).

| **Species** | **GLM** | **GBM** | **GAM** | **RF** |
| --- | --- | --- | --- | --- |
| diploids | 0.801 | 0.775 | 0849 | 0.844 |
| tetraploids | 0.771 | 0.751 | 0.787 | 0.773 |
| *R. kuepferi* s. l. | 0.781 | 0.781 | 0.796 | 0.802 |
