# Supplementary material for: Reconstructing geographical parthenogenesis: effects of niche differentiation and reproductive mode on Holocene range expansion of an alpine plant
Source: Ecol Lett. 2018 Jan 19;21(3):392–401. doi: 10.1111/ele.12908 (PMC5888191; doi:10.1111/ele.12908)
Supplement: Supplementary file 10 [file ELE-21-392-s010.docx]

**Table S3** GenBank® (http://www.ncbi.nlm.nih.gov) accession numbers for specimens used for molecular dating analyses. See (Burnier et al. 2009) for the abbreviations of the localities. All sequences were taken from (Burnier et al. 2009)as well.

| Population | ITS | *rpl*20-*rps*12 | *trn*L-*trn*F |
| --- | --- | --- | --- |
| R. kuepferi ALLA1 | EU792758 | EU792634 | EU792512 |
| R. kuepferi ALLB1 | EU792759 | EU792635 | EU792513 |
| R. kuepferi ALLC1 | EU792760 | EU792636 | EU792514 |
| R. kuepferi ALLD1 | EU792761 | EU792637 | EU792515 |
| R. kuepferi ALLD4 | EU792762 | EU792638 | EU792516 |
| R. kuepferi ALLD6 | EU792763 | EU792639 | EU792517 |
| R. kuepferi AUTH1 | EU792764 | EU792640 | EU792518 |
| R. kuepferi AUTH10 | EU792765 | EU792641 | EU792519 |
| R. kuepferi AUTH14 | EU792766 | EU792642 | EU792520 |
| R. kuepferi AUTH20 | - | EU792643 | EU792521 |
| R. kuepferi AUTH25 | EU792767 | EU792644 | EU792522 |
| R. kuepferi BOCC1 | EU792768 | EU792645 | EU792523 |
| R. kuepferi BOCC10 | EU792769 | EU792646 | EU792524 |
| R. kuepferi BRUY1 | EU792770 | EU792647 | EU792525 |
| R. kuepferi BRUY10 | EU792771 | EU792648 | EU792526 |
| R. kuepferi CAYA1 | EU792772 | EU792649 | EU792527 |
| R. kuepferi CAYB1 | EU792773 | EU792650 | EU792528 |
| R. kuepferi CAYC1 | EU792774 | EU792651 | EU792529 |
| R. kuepferi CAYD1 | EU792775 | EU792652 | EU792530 |
| R. kuepferi CAYF1 | EU792776 | EU792653 | EU792531 |
| R. kuepferi CCH11 | EU792777 | EU792654 | EU792532 |
| R. kuepferi CCH1_18 | EU792778 | EU792655 | - |
| R. kuepferi CCH1351 | EU792779 | EU792656 | EU792533 |
| R. kuepferi CCH1357 | EU792780 | EU792657 | EU792534 |
| R. kuepferi CCH33 | EU792781 | EU792658 | EU792535 |
| R. kuepferi CMAT1 | EU792782 | EU792659 | EU792536 |
| R. kuepferi CMAT11 | EU792783 | EU792660 | EU792537 |
| R. kuepferi CMAT25 | EU792784 | EU792661 | EU792538 |
| R. kuepferi COBA1 | EU792785 | EU792662 | EU792539 |
| R. kuepferi COMS1 | EU792786 | EU792663 | EU792540 |
| R. kuepferi COMS10 | EU792787 | EU792664 | EU792541 |
| R. kuepferi COMS3 | EU792788 | EU792665 | EU792542 |
| R. kuepferi COMS5 | EU792789 | EU792666 | EU792543 |
| R. kuepferi COMS8 | EU792790 | EU792667 | EU792544 |
| R. kuepferi CONG1 | EU792791 | EU792668 | EU792545 |
| R. kuepferi CONG16 | EU792792 | EU792669 | EU792546 |
| R. kuepferi CROA1 | EU792793 | EU792670 | EU792547 |
| R. kuepferi CROA20 | EU792794 | EU792671 | EU792548 |
| R. kuepferi CROB1 | EU792795 | EU792672 | EU792549 |
| R. kuepferi CSTA21 | EU792796 | EU792673 | EU792550 |
| R. kuepferi CSTB1 | EU792797 | EU792674 | EU792551 |
| R. kuepferi CSTC1 | EU792798 | EU792675 | EU792552 |
| R. kuepferi CSTD1 | EU792799 | EU792676 | EU792553 |
| R. kuepferi DEVA1 | EU792800 | EU792677 | EU792554 |
| R. kuepferi DEVC1 | EU792801 | EU792678 | EU792555 |
| R. kuepferi ERCA1 | EU792802 | EU792679 | EU792556 |
| R. kuepferi ERCA10 | EU792803 | EU792680 | EU792557 |
| R. kuepferi ERCA20 | EU792804 | EU792681 | EU792558 |
| R. kuepferi ERCB1 | EU792805 | EU792682 | EU792559 |
| R. kuepferi ERCB5 | EU792806 | EU792683 | EU792560 |
| R. kuepferi ESTB4 | EU792807 | EU792684 | EU792561 |
| R. kuepferi ESTD1 | EU792808 | EU792685 | EU792562 |
| R. kuepferi GIAU1 | EU792809 | EU792686 | EU792563 |
| R. kuepferi GIAU2 | EU792810 | EU792687 | EU792564 |
| R. kuepferi GLET1 | EU792811 | EU792688 | EU792565 |
| R. kuepferi GLET8 | EU792812 | EU792689 | EU792566 |
| R. kuepferi GOND2 | EU792813 | EU792690 | EU792567 |
| R. kuepferi GSBA1 | EU792814 | EU792691 | EU792568 |
| R. kuepferi GSBA4 | EU792815 | EU792692 | EU792569 |
| R. kuepferi GSBB9 | EU792816 | EU792693 | EU792570 |
| R. kuepferi GUEE1 | EU792817 | EU792694 | EU792571 |
| R. kuepferi HSLN4 | EU792818 | EU792695 | EU792572 |
| R. kuepferi HSLN5 | EU792819 | EU792696 | EU792573 |
| R. kuepferi IZOA1 | EU792820 | EU792697 | EU792574 |
| R. kuepferi IZOA5 | EU792821 | EU792698 | EU792575 |
| R. kuepferi IZOB2 | EU792822 | EU792699 | EU792576 |
| R. kuepferi LARC1 | EU792823 | EU792700 | EU792577 |
| R. kuepferi LARC11 | EU792824 | EU792701 | EU792578 |
| R. kuepferi LARC16 | EU792825 | EU792702 | EU792579 |
| R. kuepferi LARC3 | EU792826 | EU792703 | EU792580 |
| R. kuepferi LARC8 | EU792827 | EU792704 | EU792581 |
| R. kuepferi MOUR9 | EU792828 | EU792705 | EU792582 |
| R. kuepferi MUNT3 | EU792829 | EU792706 | EU792583 |
| R. kuepferi MUNT4 | EU792830 | EU792707 | EU792584 |
| R. kuepferi NATU1 | EU792831 | EU792708 | EU792585 |
| R. kuepferi NAVA1 | EU792832 | EU792709 | EU792586 |
| R. kuepferi NAVA6 | EU792833 | EU792710 | EU792587 |
| R. kuepferi PIEL1 | EU792834 | EU792711 | EU792588 |
| R. kuepferi PIEL3 | EU792835 | EU792712 | EU792589 |
| R. kuepferi PIEL5 | EU792836 | EU792713 | EU792590 |
| R. kuepferi PRAD1 | EU792837 | EU792714 | EU792591 |
| R. kuepferi PRAD10 | EU792838 | EU792715 | EU792592 |
| R. kuepferi PRAD6 | EU792839 | EU792716 | EU792593 |
| R. kuepferi PUNT1 | EU792840 | EU792717 | EU792594 |
| R. kuepferi PUNT8 | EU792841 | EU792718 | EU792595 |
| R. aconitifolius RAGL | EU792842 | EU792719 | EU792596 |
| R. aconitifolius RAGS1 | EU792843 | EU792720 | EU792597 |
| R. aconitifolius RAGS2 | EU792844 | EU792721 | EU792598 |
| R. aconitifolius RAMA1 | EU792845 | EU792722 | EU792599 |
| R. platanifolius RATE1 | EU792846 | EU792723 | EU792600 |
| R. platanifolius RATF | EU792847 | EU792724 | EU792601 |
| R. kuepferi ROII1 | EU792848 | EU792725 | EU792602 |
| R. kuepferi ROII16 | EU792849 | EU792726 | EU792603 |
| R. aconitifolius RPAX1 | EU792850 | EU792727 | EU792604 |
| R. platanifolius RPBO | EU792851 | EU792728 | EU792605 |
| R. platanifolius RPCA1 | EU792852 | EU792729 | EU792606 |
| R. platanifolius RPCA10 | EU792853 | EU792730 | EU792607 |
| R. platanifolius RPCN | EU792854 | EU792731 | EU792608 |
| R. platanifolius RPPP | EU792855 | EU792732 | EU792609 |
| R. seguieri RS891 | EU792856 | EU792733 | EU792610 |
| R. seguieri RSCO1 | EU792857 | EU792734 | - |
| R. seguieri RSES1 | EU792858 | EU792735 | EU792611 |
| R. kuepferi SCOK1 | EU792859 | EU792736 | EU792612 |
| R. kuepferi SCOK2 | EU792860 | EU792737 | EU792613 |
| R. kuepferi SCOK4 | EU792861 | EU792738 | EU792614 |
| R. kuepferi SIMP1 | EU792862 | EU792739 | EU792615 |
| R. kuepferi SIMP8 | EU792863 | EU792740 | EU792616 |
| R. kuepferi TENA5 | EU792864 | EU792741 | EU792617 |
| R. kuepferi TENB1 | EU792865 | EU792742 | EU792618 |
| R. kuepferi TENC1 | EU792866 | EU792743 | EU792619 |
| R. kuepferi TEND1 | EU792867 | EU792744 | EU792620 |
| R. kuepferi TENF1 | EU792868 | EU792745 | EU792621 |
| R. kuepferi TONA1 | EU792869 | EU792746 | EU792622 |
| R. kuepferi TONA10 | EU792870 | EU792747 | EU792623 |
| R. kuepferi URLE1 | EU792871 | EU792748 | EU792624 |
| R. kuepferi VARA1 | EU792872 | EU792749 | EU792625 |
| R. kuepferi VARB1 | EU792873 | EU792750 | EU792626 |
| R. kuepferi VARB5 | EU792874 | EU792751 | EU792627 |
| R. kuepferi VARC6 | EU792875 | EU792752 | EU792628 |
| R. kuepferi VARC7 | EU792876 | EU792753 | EU792629 |
| R. kuepferi VARN24 | EU792877 | EU792754 | EU792630 |
| R. kuepferi VARN5 | EU792878 | EU792755 | EU792631 |
| R. kuepferi VERA1 | EU792879 | EU792756 | EU792632 |
| R. kuepferi VERA15 | EU792880 | EU792757 | EU792633 |
