# Supplementary material for: Reconstructing geographical parthenogenesis: effects of niche differentiation and reproductive mode on Holocene range expansion of an alpine plant
Source: Ecol Lett. 2018 Jan 19;21(3):392–401. doi: 10.1111/ele.12908 (PMC5888191; doi:10.1111/ele.12908)
Supplement: Supplementary file 11 [file ELE-21-392-s011.docx]

**Appendix S1 - SUPPLEMENTARY METHODS**

***Determination of tetraploid age***

So far, no age estimate was available for the origin of tetraploid individuals of *Ranunculus kuepferi*. To exclude the possibility that tetraploids would be of younger origin than our assumed start of migration (10 k years BP), or even much older (with possible Nunatak survival during the last glacial maximum (LGM) outside the refugial area), we conducted a molecular dating based on DNA sequences from Burnier *et al.* (2009). We downloaded ITS, *rpl*20-*rps*12 and *trn*L-*trn*F sequences for 124 specimens from GenBank® (Benson *et al*. 2017) (Table S3). These included 109 *R. kuepferi* specimens representing diploid and tetraploid populations throughout the distributional range of the species, five specimens of *R. aconitifolius*, seven specimens of *R. platanifolius* and three specimens of *R. seguieri* as outgroup (Fig. S1). The molecular dating was carried out using the software BEAST v.1.8.3 (Drummond *et al.* 2012). For calibration we used the molecular dating of the genus *Ranunculus* by Emadzade and Hörandl (2011) which revealed the split of *R. kuepferi* and *R. platanifolius* in the Pliocene (mean: 3.67 MA; confidence interval 6.5 – 1 MA). We assigned a prior with a normal distribution that covered the confidence interval of Emadzade and Hörandl (2011) for the split of *R. kuepferi* from the outgroup. We used a lognormal relaxed clock, a Birth-Death prior, and the HKY substitution model was selected for all data partitions. Two independent MCMC (Markov chain Monte Carlo) searches were run for 10,000,000 generations each with one tree sampled every 1000^th^ generation. A maximum clade credibility tree was calculated in the software TreeAnnotator v.1.8.3 (Drummond *et al.* 2012) after discarding 10% of the trees as burn-in.

The maximum clade credibility tree (Fig. S1) revealed a crown group age of *R. kuepferi* s.l. of 0.15 – 1.08 MA (mean 0.53) and within the species two highly supported clades (both >0.95 posterior probability) in the southwestern Alps. One comprises the diploid and polyploid populations in the central parts of the southwestern Alps (northwards to Col d’Izoard), and hence is informative for origin of tetraploids; it has a mean age of 0.08 MA with a credibility interval of 0.01 – 0.19 MA. Hence, we can ascertain that tetraploids were already present 10,000 years before present in the southwestern Alps. They may have already originated during the late Pleistocene inside the refugial area, but there is no indication for a pre-Holocene occurrence outside this area. The other clade comprises only the geographically and genetically isolated diploid populations from Vercors (mean age 0.05 MA; credibility interval 0.001 – 0.14 MA). The other nodes of the tree have low statistical support due to the low sequence divergence in all markers, and hence no further conclusions can be drawn.

Assuming a start of simulations 10 k years BP fits the assumption that both cytotypes already had established pure populations in the southwestern Alps, although the diploids are much older. The short time span between the End of the Younger Dryas (11.9 – 10.5 k years BP) and our simulation start is regarded as negligible.

***Treeline modelling***

We applied a modelling approach in order to obtain a realistic approximation of the actual and potential upper treelines for the entire alpine arc by the use of freely available datasets and ArcGIS 10.2 software (ESRI 2011). A digital elevation model (DEM) of the Alps (horizontal resolution: 25 metres) was obtained from the European Environmental Agency. Data on current forest distribution were compiled from the Joint Research Centre forest type mapping campaign (Kempeneers *et al.* 2011) and from GIO-Land (Langanke 2013). Forest layers from these two sources were merged to produce a binary forest map. This map was further cleaned by an overlay with CORINE Land cover 2006: areas assigned to ´Natural grasslands´ (3.2.1), ´Bare rock´ (3.3.2), ´Sparsely vegetated areas´ (3.3.3) and ´Glaciers and perpetual snow´ (3.3.5) in CORINE were defined as non-forest in our map irrespective of the information provided by the Joint Research Centre or GIO-Land.

Based on this cleaned map of current forest distribution and the DEM, we assigned each 25 m-cell of the forest map the maximum elevation of all forest cells in a surrounding window of 5 km. This step should guarantee that the treeline is set to the currently realized maximum in landscapes where the forest layer is fragmented by human land use, especially by summer farming. From this maximum-elevation grid we then derived a smoothed surface of current climatic treeline elevation across the Alps (see Gehrig-Fasel *et al.* 2007 for a similar procedure). In this raster map, every cell was assigned its elevational distance to the current treeline along an imaginary vertical axis. We used the same procedure but with a surrounding window of 10 km to derive a map of the potential climatic treeline under current climatic conditions. The rationale behind was that in every area of 100 km² there should at least be one cell where the forest is at its climatically possible maximum, irrespective of treeline lowering by summer farming or topographical conditions in between. Finally, to make these maps compatible with the resolution of climatic data and CATS simulations, we resampled them to a grid size of 100 m by setting values to the maximum of all 25 m cells included in the respective 100 m cell.

Temporal change of treeline positions across the past 10 k years was modelled by, first, regressing elevational distance to the current potential treeline against current degree days (GDD, calculated following Synes & Osborne 2011) using 700,000 randomly selected sites between 1700 and 2700 m a.s.l. We used this linear regression to translate, for each 100 m x 100 m² raster cell of the study area and each time step (every interpolated 100 years’ time slice of the climate history), the difference in GDD (between current and the respective past conditions) into a new elevational distance to the potential treeline (an increase of 1 GDD at a site implies that its elevational distance to the treeline rises by *β*, the regression coefficient). The new distances were used to create a new forest map including all cells with negative distance values or zero. This forest map defined an exclusion layer for *R. kuepferi* populations, i.e. already established populations get overgrown and are removed while new ones cannot establish even if climatic conditions were suitable to the species.

We used this approach to compute the treeline for every 100 years from 8 kyr BP to 3 kyr BP. During 10 kyr BP and 8 kyr BP, we assumed the potential treeline to be higher than the realized one due to a delayed re-colonization of the Alps by forests after glacial retreat (Burga 1988; Tinner & Theurillat 2003; van der Knaap *et al.* 2005; Heiri *et al.* 2006; Brisset *et al.* 2015). We implemented the delayed recolonization process by restricting the treeline to (a maximum of) 1700 m a.s.l. in 10 kyr BP and increasing this limit up to the elevation of the climatic treeline in 8 kyr BP by linear interpolation. During the most recent 3 kyr we assumed that human land use has decreased the realized treeline in different parts of the Alps (e.g. Burga 1988; Vorren *et al.* 1993; Wick & Tinner 1997). We therefore used the difference in actual and potential treeline maps (see above) to linearly decrease the potential treeline to the actual one over this time interval. We are aware that in reality treeline decrease by human logging was abrupt and not gradual, but as we do not know establishment times of individual summer farms we used gradual lowering as averaged approximation of this patchy process across the entire Alps. Finally, we only applied the latter correction if the elevational difference between potential and actual treeline maps (see above) at a site was > 50 m, i.e. where there is (current) evidence that treelines have actually been lowered by human activities.

***Predictor variables for species distribution models***

Species distribution models (SDMs) were fit by means of seven predictor variables: three bioclimatic variables derived from monthly mean temperatures and precipitation sums; one soil variable; one variable indicating site-specific solar radiation income; and two variables describing terrain topography. The three bioclimatic variables are: maximum temperature of the warmest month (bio5); annual temperature range (bio7 which is maximum temperature of the warmest month - minimum temperature of the coldest month); and precipitation of the driest month (bio14). This set of bioclimatic variables is the same as the one used in Kirchheimer *et al.* (2016). It had been tested against a suite of alternative combinations of three variables (each with one variable representing temperature, one precipitation and one seasonal climatic differences) and was selected as the one delivering the most accurate predictions of the cytotypes’ current distribution patterns. In contrast to Kirchheimer *et al.* (2016) we did not use climatic maps from WorldClim (Hijmans *et al.* 2005) for fitting the SDMs of this study because the reconstructed climate history and the WorldClim layers differed by a clear offset, i.e. mean annual temperatures and precipitation sums between 1 k years BP and WorldClim were more different from each other than all fluctuations during the preceding 10 k years. Applying models fit with WorldClim data to the historical climatic time series would have hence delivered biased projections. We hence decided to use climatic variable values of 1 kyr BP to re-fit SDMs. We compared these re-fit models to those originally fit with WorldClim data and found that AUC values were actually even slightly higher. The new models were hence both more accurate and better compatible with the historical climatic data.

As soil variable we used percentage of calcareous bedrock per grid cell. The layer was derived from geological maps and the European Soil Database (http://eusoils.jrc.ec.europa.eu, accessed 03/11/2010). Details are described in Dullinger *et al.* (2012).

Direct and diffuse solar radiation income (in kWhm^-2^) was calculated using the Potential Incoming Solar Radiation Tool of the System for Automated Geoscientific Analyses (version 2.2.0, http://www.saga-gis.org). As input data we used the EU-DEM (EEA 2015, https://www.eea.europa.eu/data-and-maps/data/eu-dem) with a cell-size of 100 meters. Latitude, longitude and sky view factor were calculated separately and served as input parameters. Calculations were done for the days March, 20 and June, 21 on an hourly basis.

Topography of a site was described by slope inclination and curvature. The latter was calculated using the Curvature tool in the Raster surface toolset of ArcGIS 10.2 software suite (ESRI 2011) with default settings. Its output is a metric that describes the concavity/convexity of the terrain surface and hence indicates ridge vs. sink positions in the landscape.

***Demographic modelling***

In CATS, local population dynamics are driven by vital rates (germination, juvenile survival, adult survival, fecundity and survival of seeds in the seed bank) which are dependent on site suitability as measured by SDM projections. Dependence of these rates on suitability is modelled as a sigmoidal function that is constrained between zero and a maximum value (cf. Table S1). The sigmoidal functions were moreover defined by fixing their inflection points at marginal suitability (MS) values. We set marginal suitability (MS), separately for each cytotype or *R. kuepferi* s.l., to the probability of occurrence that maximizes the TSS score when used as threshold for translating probability-scaled predictions of SDMs to binary presence-absence maps (Liu *et al.* 2005). The corresponding values of the individual demographic rates were the minimum values to keep the population at a stable size. These values were found by explorative simulations, i.e. by increasing all the rates simultaneously in a stepwise fashion until the local population did not fluctuate by more than 10% over a period of 800 simulation years (following 200 years of burn-in). We note that this setup of parameters implies that demographic rates respond similar to gradients of environmental suitability which is probably not always the case (Eckhart *et al.* 2011; Villellas *et al.* 2013; Thuiller *et al.* 2014; Csergő *et al.* 2017). Nevertheless, the approach delivers a set of rate values which, in combination, have the desired property of fixing the population at the lowest possible stable value at MS. The slope parameter of the logistic functions was set to 15 as this value produced a reasonable form of the logistic function over a broad range of MS values (Dullinger *et al.* 2012).

Germination and juvenile survival were modelled as density-dependent. Density dependence was implemented by correcting the function of the respective rate to:

f_rate_cor()_ = f_rate()_ – N/C * (f_rate()_ – f_rate(MS)_)

where f_rate()_ is the original sigmoidal function, N is population size (number of individuals) at time t_-1_, C is carrying capacity and f_rate(MS)_ is the value of the original sigmoidal function at marginal suitability as defined above (see Fig. S3 in Hülber *et al.* 2016 for a graphical illustration). Where mixed populations of both cytotypes emerged during simulations, the relevant population size for density dependent effects on germination and juvenile survival (and hence the relevant carrying capacity) was the total population size of both cytotypes combined.

Our definition of f_rate_cor()_ lets population size decrease at sites with occurrence probabilities smaller than MS. However, sigmoidal functions asymptotically converge but never reach zero. To introduce strict environmental unsuitability into the model, we hence defined a further threshold of occurrence probability below which a population is assumed to decline rapidly (i.e., to ½ and ¼ for the adult and the juvenile cohort, respectively, per annual time step). For each species, we defined this threshold as the lowest occurrence probability that the SDM predicts for a site currently occupied by the respective cytotype/species according to the calibration data.

For a more detailed description of demographic models in CATS see Hülber *et al.* (2016).

***Seed dispersal***

We combined dispersal functions for four different processes likely to transport diaspores over longer distances: by wind, within the furs and guts of large herbivores, respectively, and by an unspecified long-distance dispersal (LDD) vector. For wind dispersal, we parameterized the analytical WALD kernel (Katul *et al.* 2005) with measured seed traits (see Table S1) and wind speed data from a typical high-altitude meteorological station in the Central Alps of Austria (Mt. Sonnblick, 15-min interval data from the years 2000 – 2008). Exo- and endozoochorous kernels were parameterized on the basis of correlated random walk simulations for the most frequent large herbivore in the study area, the chamois (*Rupicapra rupicapra* L.). Random walk simulations were parameterized with telemetric data taken from Fankhauser and Enggist (2004). During the random walks, seeds were attached to the animals’ fur or ingested and subsequently dropped or defecated after a time span corresponding to their surface structures and masses according to regression functions in Römermann *et al.* (2005) and Mouissie (2004). Frequency distributions of the simulated distances between points of uptake and loss were then used to construct dispersal kernels for both exo- and endozoochory. A detailed description of dispersal kernel parameterization procedures can be found in Dullinger *et al.* (2012).

The maximum dispersal distance reached by these three dispersal kernels was c. 10 km. To cover rare LDD events exceeding 10 km in dispersal distance, we additionally assumed that 0.1% of each population’s seed yield was distributed randomly within a radius of 50 km. This proportion is lower than the estimated 1% in Vittoz and Engler (2007), but, in exchange, the kernel width (50 km) is large. We assumed that dispersal over such large distances must have occasionally occurred because isolated populations of *R. kuepferi* can be found in the Apennines on the island of Corsica today (Cosendai & Hörandl 2010). The remaining seed yield (i.e. 99.9%) was partitioned among the three dispersal kernels (1-5% exo-, 1-5% endozoochorous, rest wind; exact number selected randomly for each year and site/population) assuming that wind is the ‘standard’ vector (Müller-Schneider 1986).

**REFERENCES**

Asker, S.E. & Jerling, L. (1992). *Apomixis in Plants*. CRC Press, Boca Raton, FL.

Benson, D.A., Cavanaugh, M., Clark, K., Karsch-Mizrachi, I., Lipman, D.J., Ostell, J. *et al.* (2017). GenBank. *Nucleic Acids Res*, 45, D37-D42.

Brisset, E., Guiter, F., Miramont, C., Revel, M., Anthony, E.J., Delhon, C. *et al.* (2015). Lateglacial/Holocene environmental changes in the Mediterranean Alps inferred from lacustrine sediments. *Quaternary Sci Rev*, 110, 49-71.

Burga, C.A. (1988). Swiss Vegetation History during the Last 18000 Years. *New Phytol*, 110, 581-602.

Burnier, J., Buerki, S., Arrigo, N., Küpfer, P. & Alvarez, N. (2009). Genetic structure and evolution of Alpine polyploid complexes: Ranunculus kuepferi (Ranunculaceae) as a case study. *Mol Ecol*, 18, 3730-3744.

Cosendai, A.-C. & Hörandl, E. (2010). Cytotype stability, facultative apomixis and geographical parthenogenesis in Ranunculus kuepferi (Ranunculaceae). *Ann Bot-London*, 105, 457-470.

Csergő, A.M., Salguero-Gómez, R., Broennimann, O., Coutts, S.R., Guisan, A., Angert, A.L. *et al.* (2017). Less favourable climates constrain demographic strategies in plants. *Ecol Lett*, 20, 969-980.

Drummond, A.J., Suchard, M.A., Xie, D. & Rambaut, A. (2012). Bayesian Phylogenetics with BEAUti and the BEAST 1.7. *Mol Biol Evol*, 29, 1969-1973.

Dullinger, S., Gattringer, A., Thuiller, W., Moser, D., Zimmermann, N.E., Guisan, A. *et al.* (2012). Extinction debt of high-mountain plants under twenty-first-century climate change. *Nat Clim Change*, 2, 619-622.

Eckhart, V.M., Geber, M.A., Morris, W.F., Fabio, E.S., Tiffin, P. & Moeller, D.A. (2011). The geography of demography: Long-term demographic studies and species distribution models reveal a species border limited by adaptation. *Am Nat*, 178, S26-S43.

Emadzade, K. & Hörandl, E. (2011). Northern Hemisphere origin, transoceanic dispersal, and diversification of Ranunculeae DC. (Ranunculaceae) in the Cenozoic. *J Biogeogr*, 38, 517-530.

ESRI (2011). ArcGIS Desktop: Release 10. Redlands,CA: Environmental Systems Research 1999-2011.

Fankhauser, R. & Enggist, P. (2004). Simulation of alpine chamois Rupicapra r. rupicapra habitat use. *Ecol Model*, 175, 291-302.

Gehrig-Fasel, J., Guisan, A. & Zimmermann, N.E. (2007). Tree line shifts in the Swiss Alps: Climate change or land abandonment? *J Veg Sci*, 18, 571-582.

Heiri, C., Bugmann, H., Tinner, W., Heiri, O. & Lischke, H. (2006). A model-based reconstruction of Holocene treeline dynamics in the Central Swiss Alps. *J Ecol*, 94, 206-216.

Hijmans, R.J., Cameron, S.E., Parra, J.L., Jones, P.G. & Jarvis, A. (2005). Very high resolution interpolated climate surfaces for global land areas. *Int J Climatol*, 25, 1965-1978.

Hülber, K., Wessely, J., Gattringer, A., Moser, D., Kuttner, M., Essl, F. *et al.* (2016). Uncertainty in predicting range dynamics of endemic alpine plants under climate warming. *Global Change Biol*, 22, 2608-2619.

Katul, G.G., Porporato, A., Nathan, R., Siqueira, M., Soons, M.B., Poggi, D. *et al.* (2005). Mechanistic analytical models for long-distance seed dispersal by wind. *Am Natt*, 166, 368-381.

Kempeneers, P., Sedano, F., Seebach, L., Strobl, P. & San-Miguel-Ayanz, J. (2011). Data Fusion of Different Spatial Resolution Remote Sensing Images Applied to Forest-Type Mapping. *Ieee T Geosci Remote*, 49, 4977-4986.

Kirchheimer, B., Schinkel, C.C.F., Dellinger, A.S., Klatt, S., Moser, D., Winkler, M. *et al.* (2016). A matter of scale: apparent niche differentiation of diploid and tetraploid plants may depend on extent and grain of analysis. *J Biogeogr*, 43, 716-726.

Langanke, T. (2013). GIO land (GMES/Copernicus initial operations land) High Resolution Layers (HRLs) – summary of product specifications; Version 6 of 2013-07-08. European Environmental Agency, Copenhagen. Available at: http://land.copernicus.eu/user-corner/publications/gio-land-high-resolution-layers/view/.

Liu, C.R., Berry, P.M., Dawson, T.P. & Pearson, R.G. (2005). Selecting thresholds of occurrence in the prediction of species distributions. *Ecography*, 28, 385-393.

Mouissie, A.M. (2004). Seed dispersal by large herbivores: Implications for the restoration of plant biodiversity (Doctoral thesis). University of Groningen.

Müller-Schneider, P. (1986). *Verbreitungsbiologie der Blütenpflanzen Graubündens*. Veröffentlichungen des Geobotanischen Institutes der ETH, Stiftung Rubel, Zürich.

Römermann, C., Tackenberg, O. & Poschlod, P. (2005). How to predict attachment potential of seeds to sheep and cattle coat from simple morphological seed traits. *Oikos*, 110, 219-230.

Richards, J. A. (1997). Plant breeding systems. 2nd ed. Chapman & Hall, London.

Synes, N.W. & Osborne, P.E. (2011). Choice of predictor variables as a source of uncertainty in continental-scale species distribution modelling under climate change. *Glob. Ecol. Biogeogr.*, 20, 904-914.

Thuiller, W., Münkemüller, T., Schiffers, K.H., Georges, D., Dullinger, S., Eckhart, V.M. *et al.* (2014). Does probability of occurrence relate to population dynamics? *Ecography*, 37, 1155-1166.

Tinner, W. & Theurillat, J.P. (2003). Uppermost limit, extent, and fluctuations of the timberline and treeline ecocline in the Swiss Central Alps during the past 11,500 years. *Arct Antarct Alp Res*, 35, 158-169.

van der Knaap, W.O., van Leeuwen, J.F.N., Finsinger, W., Gobet, E., Pini, R., Schweizer, A. *et al.* (2005). Migration and population expansion of Abies, Fagus, Picea, and Quercus since 15000 years in and across the Alps, based on pollen-percentage threshold values. *Quaternary Sci Rev*, 24, 645-680.

Villellas, J., Morris, W.F. & Garcia, M.B. (2013). Variation in stochastic demography between and within central and peripheral regions in a widespread short-lived herb. *Ecology*, 94, 1378-1388.

Vittoz, P. & Engler, R. (2007). Seed dispersal distances: a typology based on dispersal modes and plant traits. *Bot Helv*, 117, 109-124.

Vorren, K.-D., Mørkved, B. & Bortenschlager, S. (1993). Human impact on the Holocene forest line in the Central Alps. *Veg Hist Archaeobot*, 2, 145-156.

Wick, L. & Tinner, W. (1997). Vegetation changes and timberline fluctuations in the central alps as indicators of Holocene climatic oscillations. *Arctic Alpine Res*, 29, 445-458.
