# Supplementary material for: Reconstructing geographical parthenogenesis: effects of niche differentiation and reproductive mode on Holocene range expansion of an alpine plant
Source: Ecol Lett. 2018 Jan 19;21(3):392–401. doi: 10.1111/ele.12908 (PMC5888191; doi:10.1111/ele.12908)
Supplement: Supplementary file 13 [file ELE-21-392-s013.docx]

**Appendix S2 - SUPPLEMENTARY MOVIE**

To comply with storage restrictions upon submission the video is provided under the following link:

http://131.130.33.15/rankuepf/rkuepf.mpeg

Upon acceptance a compressed version of the video will be uploaded directly onto the journal homepage.
